# Probing the Mineral–Water Interface with Nonlinear Optical Spectroscopy

**DOI:** 10.1002/anie.202003085

**Published:** 2020-12-09

**Authors:** Ellen H. G. Backus, Jan Schaefer, Mischa Bonn

**Affiliations:** ^1^ Max Planck Institute for Polymer Research Ackermannweg 10 55128 Mainz Germany; ^2^ Department of Physical Chemistry University of Vienna Währinger Strasse 42 1090 Vienna Austria

**Keywords:** alumina, calcium fluoride, nonlinear optics, silica, vibrational spectroscopy

## Abstract

The interaction between minerals and water is manifold and complex: the mineral surface can be (de)protonated by water, thereby changing its charge; mineral ions dissolved into the aqueous phase screen the surface charges. Both factors affect the interaction with water. Intrinsically molecular‐level processes and interactions govern macroscopic phenomena, such as flow‐induced dissolution, wetting, and charging. This realization is increasingly prompting molecular‐level studies of mineral–water interfaces. Here, we provide an overview of recent developments in surface‐specific nonlinear spectroscopy techniques such as sum frequency and second harmonic generation (SFG/SHG), which can provide information about the molecular arrangement of the first few layers of water molecules at the mineral surface. The results illustrate the subtleties of both chemical and physical interactions between water and the mineral as well as the critical role of mineral dissolution and other ions in solution for determining those interactions.

## Graphical abstract

The interaction between minerals and water is relevant for a wide range of geological, atmospheric, environmental, and electro(photo‐)chemical systems. This Review highlights the developments made through studying a variety of mineral–water interfaces by nonlinear optical spectroscopy. These methods provide molecular‐level details about the organization of water in the direct vicinity of the interface.

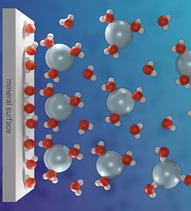

## 1 Introduction

Mineral–water interfaces are ubiquitous, spanning from sand in seawater to rain on rocks or windowpanes. Biomineralization occurs in the presence of water,^[1]^ such as the formation of teeth in saliva. Furthermore, photocatalytic reactions that dissociate water into its elements can occur at the interface between certain minerals and water. In the troposphere, water droplets nucleate on mineral particles, thereby creating aqueous mineral dust aerosols.^[2]^ The interaction of water with minerals is also a major pathway for chemical reactions occurring in nature.^[3]^ The ability of water to dissolve and precipitate minerals, thus driving their distribution on Earth through rivers and oceans, is clear. The role of (interfacial) water in driving geological processes even within the Earth's crust has recently also been emphasized.^[4]^

Mineral surfaces typically carry charges originating, for example, from ion substitution in the lattice when the mineral is crystallized from its melt. In contact with water, the charged state of the mineral surface could change as a result of surface reactions. For oxides, the surface charge can originate from protonation or deprotonation of groups that terminate the surface. An example of this class of molecular groups are silanols (Si‐O‐H) that terminate the silica surface. The pH value and ionic strength of the aqueous solution in contact with the surface determine the sign and degree of charge. For minerals based on ionic lattices, differences in the dissolution rates of the different ionic constituents can give rise to a surface charge. One example of this is CaF_2_ at acidic pH values. The ready dissolution of fluoride ions compared to calcium ions gives rise to a positively charged surface.^[5]^

The charge at the mineral surface affects the interfacial water structure, which has consequences for the physicochemical properties of the interface and, in turn, affects mineral dissolution. This recursive interplay between the mineral surface and water, as well as the multitude of chemical and physical processes occurring at the interface make this system a challenge for the experimental and modeling communities alike.

The widespread relevance of the water–mineral interface has prompted many efforts aimed at obtaining a better understanding of these interfaces. Much of this work has shown that interfacial water behaves very differently than bulk water. A water molecule in the liquid bulk is, on average, tetrahedrally coordinated, donating two and accepting two hydrogen bonds. Hydrogen bonding, and the collective effects resulting from the propagating hydrogen‐bond network, are key in determining the properties of bulk liquid water.^[6]^ At an interface, the hydrogen‐bonded water network is interrupted, and non‐tetrahedrally coordinated water molecules become more prevalent. At the macroscopic level, the termination of the bulk hydrogen‐bonding network gives rise to, for example, the anomalously high surface tension of the free water surface and the anomalous drop in the dielectric function from about 80 in the bulk to about 3 at interfaces.^[7]^ The complexity of aqueous interfaces is further increased by the fact that their surface is neither flat nor characterized by a uniform charge density, but is instead heterogeneous in both morphology and charge distribution.

Many continuum models exist to describe the interaction between a charged mineral surface and an electrolyte solution. The Gouy–Chapman model, based on the Poisson–Boltzmann equation, is possibly the most widespread. This mean‐field description assumes, for the surface, the water, and ions dissolved in the water, respectively, that: 1) the surface is homogeneously charged, and spatially perfectly sharp, 2) ions are point charges, interacting only through Coulomb interactions; and 3) water is a homogeneous dielectric continuum.

None of these assumptions is rigorously valid, and their shortcomings are most apparent on short length scales. Over longer length scales, these theories are quite reliable, since electrostatic interactions are long‐range and local details average out. It is evident, however, that molecular‐level processes underlie the most important processes and properties—even apparently macroscopic ones such as wetting—occurring at mineral–water interfaces. Therefore, it is important to understand the interfacial water structure of water–mineral interfaces.

From the perspective of modeling, mineral–water interfaces pose a major challenge. Classical molecular dynamics (MD) and ab initio MD (AIMD) approaches work well for bulk phases, but an accurate description of the interaction with surfaces remains a major challenge—even using ab initio approaches. As such, computational studies are only slowly beginning to provide realistic molecular‐level models of interface reactions of aqueous mineral solutions as well as structures consistent with experimental results. Moreover, simulations could play an important role in resonance assignments. As this Review focusses on experimental work, no simulation work is explicitly discussed.

From the experimental side, substantial progress in our understanding of solid–liquid interfaces has been made by using various techniques. Although this Review is limited to nonlinear optical probes of mineral–water interfaces, several important breakthroughs have been achieved using other techniques: atomic force microscopy, for example, has revealed the layering of water at mineral surfaces;^[8]^ various synchrotron‐based X‐ray approaches, including X‐ray absorption, diffraction, and X‐ray photoelectron spectroscopy, have been employed to shed light on the mineral surface charge, surface chemical composition, and the ion distribution near the surface, as well as the organization of water.^[9]^ These techniques can probe mineral interfaces in real‐space (scanning force microscopy) or *k*‐space (X‐ray spectroscopy) on molecular length scales, but are both potentially rather invasive. Non‐invasive optical spectroscopy, and in particular vibrational spectroscopy, can, therefore, nicely complement these methods. Linear vibrational spectroscopic methods have molecular specificity but are not sensitive to the interface region.

Nonlinear optics typically involve the frequency conversion of optical fields by a nonlinear interaction with a material or its surface. In vibrational sum‐frequency generation (SFG) spectroscopy, an infrared (IR) and a visible (Vis) pulsed laser beam are overlapped in space and time at an interface, thereby generating photons at the sum‐frequency of the two incident frequencies. A crucial selection rule for SFG is that the centrosymmetry must be broken, which intrinsically happens at the interface between two media. Moreover, when the surface is charged, that charge will align water molecules near the surface, further breaking the symmetry. Vibrational information can be obtained by tuning the IR frequency with a vibrational mode.^[10]^ Second‐harmonic generation (SHG) is a degenerate case of SFG in which only one laser beam is used, and photons at twice the frequency of the incident field are generated. Although SHG, a nonresonant second‐order optical process, is nonselective to particular molecular or atomic species, SFG may report on specific vibrational resonances, for example, the O‐H stretch vibrations of water. This may give rise to different physical mechanisms to explain the corresponding nonlinear responses and, therefore, differ in their interpretation. In addition to this, both techniques can be employed in static and time‐resolved manners, which allow information to be retrieved on the structure and dynamics of the system, respectively. Both techniques can also be used in a scattering geometry, thereby providing access to the surfaces of nano‐ and microparticles.^[11]^ Here, we limit ourselves to experimental nonlinear optical spectroscopy on planar interfaces.

Both SHG and SFG have been used to study water–mineral interfaces, and have proven their strength in answering some of the questions raised above. In SFG, the IR frequency is typically tuned to be resonant with the O‐H stretching mode of water. The intensity of the signal in the O‐H stretch region is a direct measure of the degree of interfacial water alignment. The sign of the nonlinear optical susceptibility reflects the absolute orientation (pointing towards or away from the surface, on average). The spectral response provides information about the hydrogen‐bonding strength of interfacial water molecules. These properties make nonlinear spectroscopy a powerful tool for the study of interfacial water near mineral surfaces.^[12]^ By using different polarization combinations for the two incident (IR and Vis) and the outgoing SFG beams, for example, ssp (s: SFG/SHG, s: Vis, p: IR) or ppp (all beams p polarized), different tensor elements of the optical susceptibility are addressed.^[13]^ The relative intensity of the signals acquired under different polarization combinations reports the preferential orientation (distribution) of the molecules at the interface, after correction for Fresnel factors. However, to obtain information on the orientation, an angular distribution has to be assumed. In this context, the combination of nonlinear optics with (ab initio) molecular dynamics simulations—which can provide such distributions—is very powerful, but outside the scope of this Review.

## 2 General Considerations for Nonlinear Optical Probes of the Mineral–Water Interface

**Figure 1:**
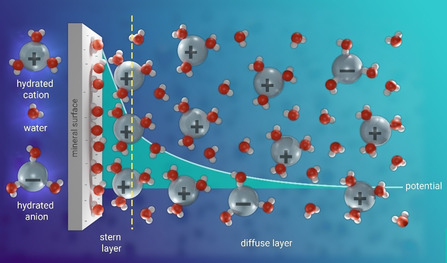
Schematic representation of the organization of ions and water at the interface between an aqueous electrolyte solution and a negatively charged mineral surface. Also plotted is the surface potential as described by the Gouy–Chapman–Stern model.

The interfacial region probed by SFG/SHG consists of those water layers that differ from the bulk structure, often referred to as the electric double layer (EDL).^[12]^ The EDL can be loosely defined as the interfacial structure of water and counterions that appears at the charged surface of any material and consists of the near‐surface Stern layer and the diffuse layer (Figure 1). In the Gouy–Chapman–Stern description, the surface potential decays linearly in the stern layer, while it decays exponentially in the diffuse layer according to *φ*(*z*)=*φ*_0_exp(−*z*/*λ*_Debye_), with *z* being the distance to the surface and *φ*_0_ the potential at *z*=0. Generally speaking, the thickness of the electric double layer can be tuned by varying the concentration of ions in the bulk liquid; the ions in solution can screen the surface charge and thereby alter the EDL by changing the decay length of the associated surface potential—known as the Debye length *λ*_Debye_. The Debye length essentially determines the spatial range over which the symmetry is broken, and thereby the depth probed by SHG and SFG. Salt‐dependent studies potentially provide insights into the charge distribution across the EDL and the decay of the surface potential associated with it. For silica, this approach has been used with both SFG^[14]^ and SHG^[15]^ methods.

The EDL can also be modified by changing the surface charge of a mineral in contact with water by varying the bulk pH value. In the case of silica, for example, the pH value determines the fraction of deprotonated surface silanol groups. By varying the pH value at a fixed, rather high background electrolyte concentration (>0.1 m), the nonlinear optical SHG/SFG response is primarily sensitive to changes in the layers close to the surface, which may report on the surface charge density and associated surface potential either at the surface plane (*φ*_0_) or at the outer Helmholtz plane (*φ*_ζ_), depending on the background electrolyte concentration (see Refs. [14a, 16] for SHG and [16e, 17] for SFG).

It is important to note that the symmetry breaking necessary for the generation of SHG/SFG signals can have two distinct origins. Firstly, the presence of an interface, independent of its charged state, causes symmetry breaking per se: at the interface, the local water structure is modified because of the different hydrogen‐bonding interaction with the interface than with water. If the surface is charged, the charge can cause preferential alignment of water, further breaking the symmetry. These effects can roughly be termed *χ* ^(2)^ effects, as they affect the second‐order nonlinear optical susceptibility. Secondly, the presence of a static field can give rise to a *χ* ^(3)^ response. Simply stated, the field can polarize otherwise (bulk‐like) randomly oriented water molecules, and break the symmetry in that manner, over a length scale determined by the Debye length. The magnitude of the relative contributions from the *χ* ^(2)^ versus *χ* ^(3)^ responses depends on the details of the system (surface charge, specific interactions between water and the surface, electrolyte concentration, etc.) and how it is probed.

**Figure 2:**
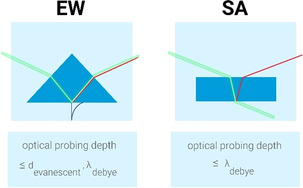
Schematic representation of the evanescent wave (EW) and steep angle (SA) geometry. In the EW geometry, the optical penetration depth of the incident beams is limited to the evanescent field, and the effective probing depth is determined by the evanescent depth and the Debye length. In the SA geometry, the probing depth is only determined by the Debye length.

Specifically, for all second‐order spectroscopy techniques, the optical limit for the probing depth critically depends on the geometry of the involved beams, as it is an interplay between coherence length and Debye length.[^16e^, ^18^] Figure 2 shows schematic pictures of the two typical geometries used in nonlinear surface spectroscopy experiments. In the evanescent wave (EW) geometry, the optical fields are enhanced in the near‐surface region but decay exponentially with distance from the surface (with decaying length *d*_evanescent_), thereby limiting the probing depth. At high salt concentrations the Debye length *λ*_Debye_ could be shorter than *d*_evanescent_, in which case *λ*_Debye_ determines the probing depth. In steep‐angle (SA) reflection geometry, the penetration depth is limited by the absorption depth of the IR beam, which, for the O‐H stretch of water, occurs in the micrometer range. However, *λ*_Debye_ often determines the probing depth of the optical signal. In the EW geometry, the evanescent wave gives rise to a penetration depth of tens of nanometers, which may be exceeded by the Debye length of the probed interface. As a result, EW‐SFG is more surface‐specific than the SA analogue, as it preferentially reports on the signal contributions from the individual layers in the near‐surface region.

## 3 Silica

Owing to its favorable optical properties and high abundance, the silica–water interface is one of the most extensively studied buried interfaces. Silica, as a prototypical mineral can be chemically altered in terms of both the surface charge as well as the interfacial charge distribution. The surface charge of silica is tunable over a large pH range since the point of zero charge (pzc) is as low as about pH 2.

### 3.1 Counterion Dependence

Pioneering studies in the field of nonlinear spectroscopy of the silica–water interface were presented by Eisenthal and co‐workers in 1992.^[14a]^ In this study, they investigated the silica surface in contact with lithium chloride and sodium chloride solutions by using SHG spectroscopy with a EW geometry. They observed that independent of the cationic species, the SHG intensity decreases as the ion concentration increases from 0.01 to 0.1 m. Since the SHG signal reflects the number of polarized and reoriented water molecules induced by the electric field, in association with the surface potential, this observation was opposite to what they expected based on the prior assumption that salt promotes the deprotonation of silanol and thus increases the silica surface charge. In contrast, the observed relationship between ion concentration and SHG response was rationalized with the Gouy–Chapman equation for the surface potential, which predicts a decrease in the signal intensity with an increasing ion concentration *c*.

An SFG study by Chou and co‐workers showed a similar trend for the O‐H stretch vibrational response of aqueous alkali chloride solutions with the ssp polarization combination. They interpreted the results as an ion‐induced perturbation of the interfacial water network.^[14b]^ Based on the observed concentration sensitivity of the SFG signal in the order K^+^>Li^+^>Na^+^, they concluded a corresponding ion‐dependent degree of perturbation. Since this trend is not monotonic with ion size, they interpreted this observation as a combination of two counteracting effects associated with the hydration radii of the different cations: Hydration water may replace interfacial water but also promote silanol dissociation, which de‐ and increases the SFG response, respectively. Additionally, they realized that the SFG response consists of two spectroscopic features in the O‐H stretching region of hydrogen‐bonded groups (at ca. 3200 cm^−1^ and 3400 cm^−1^). Independent of the salt species, the low‐frequency band appeared to be more affected by the variation of the salt concentration. Based on studies on α‐quartz in water with various polarization combinations,^[19]^ Shen and co‐workers argued that the low‐frequency band is associated with water at (coupled) vicinal silanol groups that, when dissociated, generate a higher local surface charge density and, therefore, high SFG intensity. They concluded that both bands stem from the region close to the surface, but the stronger concentration dependency of the low‐frequency band reflects the preferential ion‐induced perturbance of the areas of high surface charge density.

Jena and Hore performed an SFG study under EW geometry with NaCl solutions using the ssp and sps polarization combination.^[14c]^ They found two to three features in the O‐H stretching region of hydrogen‐bonded OH groups with different relative intensities for ssp and sps, respectively. They argued that as the frequency is decreased, the O‐H stretching band reflects more highly coordinated water molecules and more symmetric over asymmetric stretching modes. In support of previous studies by Chou and co‐workers,^[14b]^ they found that as the NaCl concentration was increased, the overall SFG response and the ratio between low‐ and high‐frequency bands decreased. Additionally, the sps/ssp ratio of the two main features decreases as well. In contrast to the previous interpretation,^[14b]^ they concluded that two main species of interfacial water exist, one close to the interface with lower coordination and the other further away from the interface that is more highly coordinated. They argued that the surface charge gets screened as the salt concentration increases, which results in a thinner surface layer accompanied by a relative reduction in the number of highly coordinated water molecules further away from the surface. Based on the polarization ratios, they additionally concluded that, as the concentration increases, the average tilt angle changes from 70° to 55°, thus towards a dipole orientation more aligned with the surface normal.

**Figure 3:**
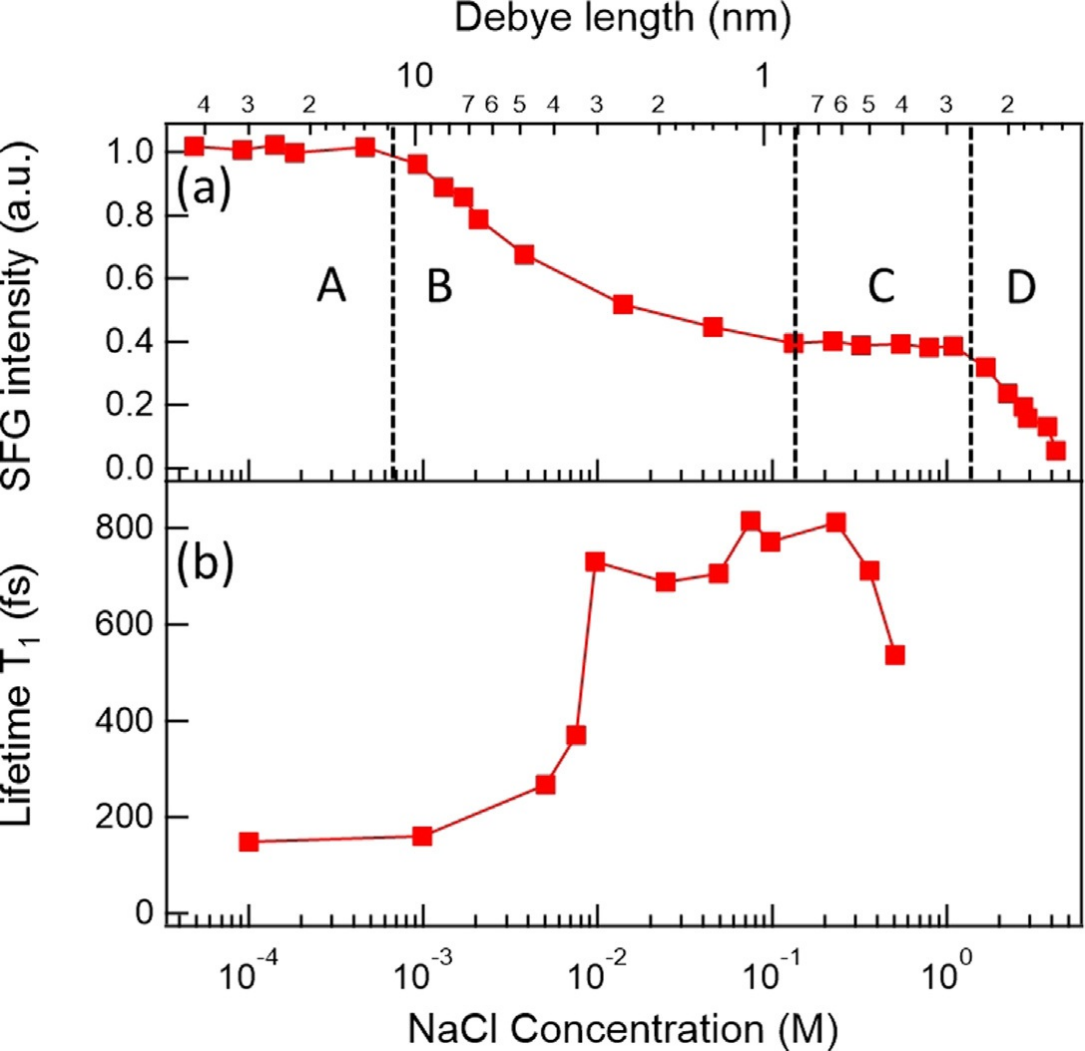
a) Integrated O‐H stretch (ssp) SFG signal of the silica–water interface as a function of NaCl concentration. The top axis presents the theoretical Debye length, calculated based on the Gouy–Chapman model. Adapted from Ref. [20] with permission. Copyright 2011 American Chemical Society. b) Vibrational lifetimes of the O‐H stretch (ssp) SFG signal of the same system, also as a function of NaCl concentration. Adapted from Ref. [22] with permission. Copyright 2011 American Chemical Society.

In 2011, Hore and co‐workers performed additional SFG experiments with ssp polarization of the silica–water system through a systematic approach involving changing the ionic strength over a broad range from sub‐mm up to the dissolution limit.^[20]^ Similar to the previous results, they found an overall monotonically decreasing signal as the NaCl concentration increased. However, they were able to distinguish between four different concentration regimes, which are presented in Figure 3 a and briefly summarized below, together with the provided conclusion:

- *c*<0.5 mm: The SFG response is insensitive to variation of the salt concentration: Ions may promote silanol deprotonation, but also screen the further distant water layers from that charge. This may give rise to a balance between an increase in the surface layer (SL) response (described by the *χ* ^(2)^ response) and a decrease in the diffuse layer (DL) response (described by the so‐called *χ* ^(3)^ response) at this low concentration. See for example, Refs. [14e, 16e, 21] for a more extended discussion about an *χ* ^(2)^ versus *χ* ^(3)^ response.
- 0.5 mm<*c*<100 mm: The SFG signal decreases upon adding salt: The ions increasingly screen the surface charges, which gives rise to a decreasing *χ* ^(3)^ contribution.
- 0.1 m<*c*<1 m: A second plateau reflects the insensitivity of the SFG response towards an increasing salt concentration, which is interpreted as a *χ* ^(2)^‐dominated signal, with both *χ* ^(2)^ and *χ* ^(3)^ contributions remaining constant. In the physical model, this regime represents the transition from the Gouy–Chapman to the Stern description of the interface.
- *c*>1 m: The SFG signal continues to decrease: In this high concentration regime, the interfacial hydrogen‐bonding environment gets perturbed by the ions, inducing a less ordered interfacial water structure.

In complementary work, Borguet and co‐workers performed a time‐resolved SFG study of the same systems, also under EW geometry and with ssp polarization, which is summarized in Figure 3 b. They found that the vibrational lifetime of the H‐bonded O‐H stretch at low ion concentrations is comparable to that of bulk water (ca. 200 fs), in line with the results from Hore and co‐workers that showed that mainly the bulk is detected in the SFG at low salt concentrations. In contrast, they observed substantially longer lifetimes (ca. 700 fs) for higher ion concentrations (*c*>0.01 m), where the SFG response is more surface‐specific.^[22]^ These lifetime measurements indicate that a salt concentration of about 10 mm is sufficient to suppress the bulk contribution. A very recent time‐resolved SFG study combined with ab initio DFT‐based molecular dynamics simulations revealed that ion adsorption at the silica surface can effectively change the hydrophobicity of the surface, thereby leading to a strong reduction in the lifetime of the vibration.^[23]^

Overall, these studies highlight the role of the *χ* ^(3)^ contribution to the nonlinear response of water at charged interfaces. They show that for a low salt concentration (*c*<10 mm), the (long‐ranging) field‐induced water response may be dominating, and the underlying structure and dynamics of those water layers behave like the bulk phase. On the basis of their findings, a follow‐up study, as well as with ssp polarization, was reported by Backus and co‐workers in 2017.^[14e]^ The findings demonstrate that the SFG signal is not constant across regime A but, in contrast to regime B–D, decreases as the salt concentration decreases, which becomes evident, especially when using the SA geometry. This trend was predicted by Gonella et al., who developed a model that invokes charge screening and optical interference to determine the SFG response.^[16e]^ In an SHG study under EW geometry carried out by Eisenthal and co‐workers, a similar trend was observed, which was found to be independent of the polarization combination (p‐in or s‐in, all‐out) or the ion size.^[15]^ However, to achieve decent agreement between their experiments and the model mentioned above, a dramatic and exotic adjustment of the relative permittivity from 80 for bulk water to 30 for the diffuse layer was necessary. Additionally, further changes in the surface charge and/or Stern layer charge were also necessary. A follow‐up study from 2019 showed that the level of the plateau at high concentration depends on the size of both the cation and anion.^[24]^ This was interpreted as ion‐specific Stern‐layer properties among the investigated alkali halides.

**Figure 4:**
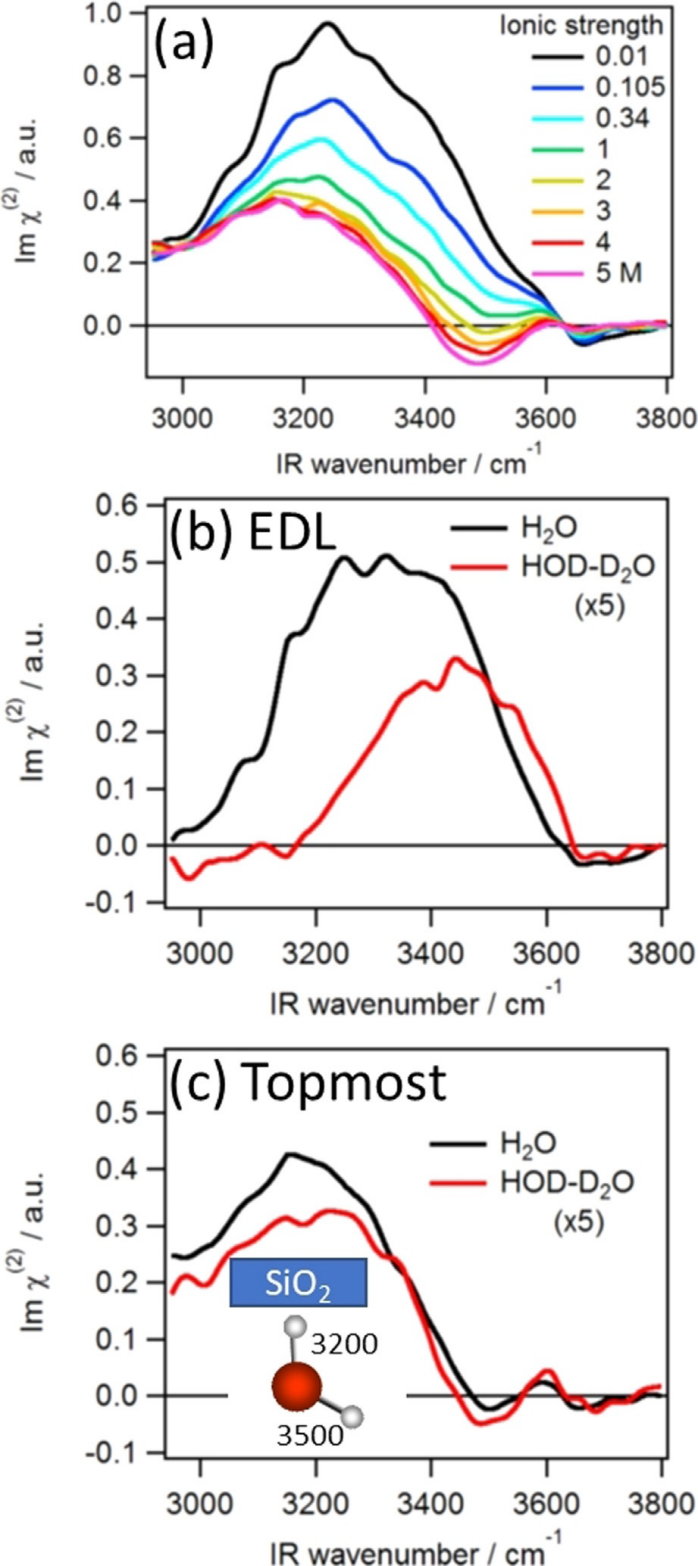
a) Phase‐resolved SFG spectrum of silica–H_2_O at pH 12 as a function of salt concentration. b) Difference spectrum between 0.01 m and 1 m for H_2_O and isotopically diluted water. c) 2 m spectrum with H_2_O and isotopically diluted water. HOD‐D_2_O means a sample with the ratio H_2_O/HOD/D_2_O=1:8:16. Reprinted from Ref. [14g] with permission. Copyright 2018 American Chemical Society.

In 2018, Tahara and co‐workers performed phase‐resolved ssp measurements of the silica–water interface under conditions of high surface charge (pH 12), in which they observed a similar dependence on the ion concentration.^[14g]^ Additionally, they were able to separate the contributions of DL and SL water species to the overall ion‐dependent SFG response. As illustrated in Figure 4 a, they observed that the signal decreases with ionic strength between 10 mm and 1 m NaCl and is saturated between 2 m and 5 M. As a consequence, they considered the difference spectrum between 10 mm and 1 m to represent the DL part and the high concentration spectra as the SL part. The DL spectrum consists of two broad features at around 3200 and 3400 cm^−1^. Since the 3200 cm^−1^ part vanished upon isotopic dilution (Figure 4 b), which is an indicator of vibrational coupling, the DL response was characterized to be bulk‐like. In contrast, the SL spectrum (Figure 4 c) was insensitive to isotopic dilution, which suggested water species that are not bulk‐like. Furthermore, the SL spectrum consists of two features: One pronounced positive band at 3200 cm^−1^, indicating an H‐up orientation with strong hydrogen bonds to the silica surface, and one weak negative band at 3500 cm^−1^, suggesting H pointing down with weak hydrogen bonds. Based on that observation, they concluded that the topmost water layer oriented with one hydroxy group hydrogen bonding to the surface and the other one pointing down towards the bulk water.

### 3.2 pH Dependence

**Figure 5:**
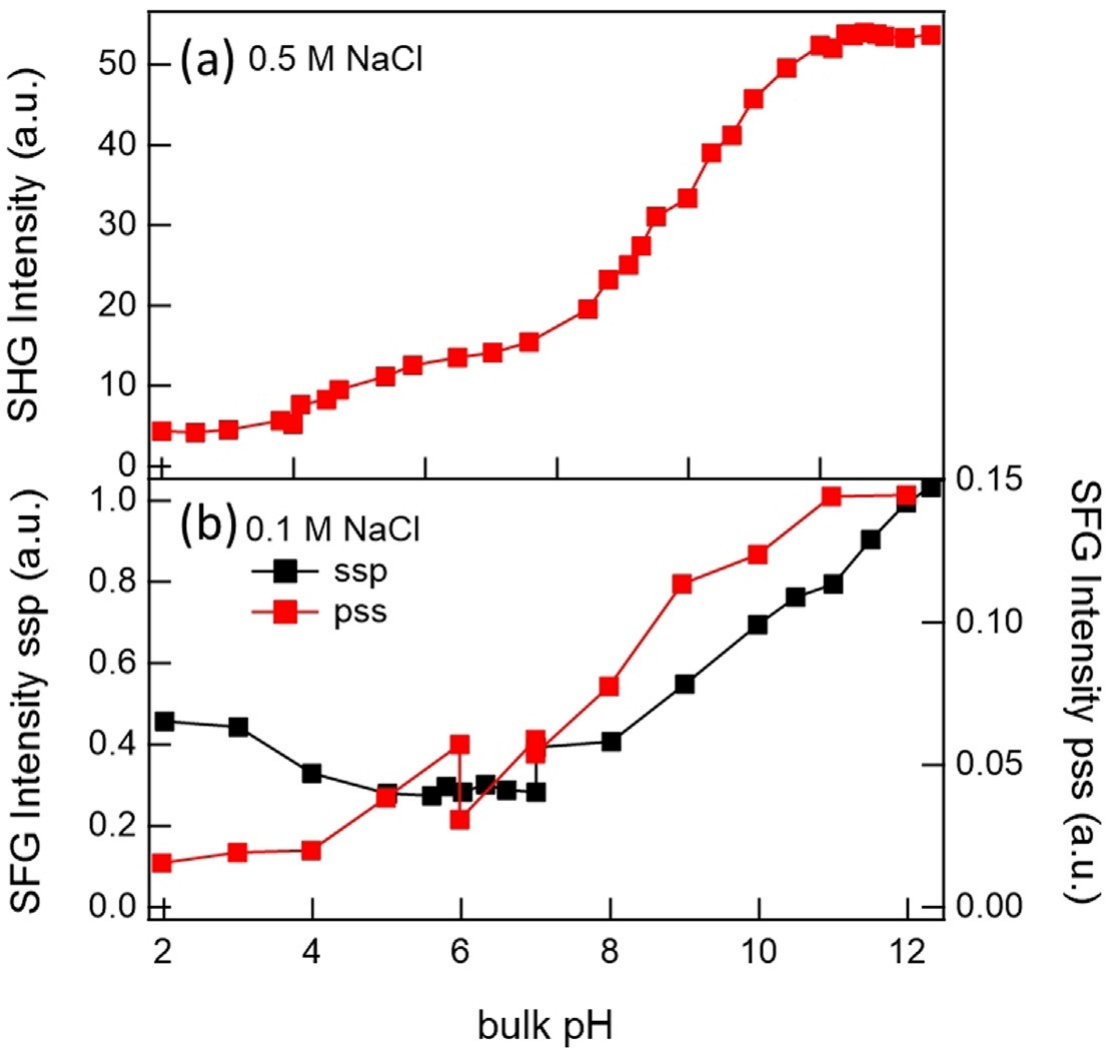
a) Variation of the SH electric field of the silica–water interface with a changing bulk pH value at a constant electrolyte concentration (*c*(NaCl) = 0.5 m). Adapted from Ref. [14a], with permission. Copyright 1991, Elsevier. b) Integrated O‐H stretch ssp versus ppp polarization SFG signal (EW geometry) as a function of pH value with 100 mm background electrolyte (NaCl). Adapted from Ref. [17c] with permission. Copyright 2017 American Chemical Society.

The study presented by Eisenthal and co‐workers in 1992 mentioned above was also the first attempt to further the understanding of the acid–base chemistry of the silica–water interface using nonlinear optical spectroscopy.^[14a]^ By employing SHG spectroscopy with EW geometry, they recorded a surface titration curve with a 0.5 m NaCl background electrolyte. As depicted in Figure 5 a, they observe a monotonic increase in the SHG response as the pH value was increased from 2 to 14. The titration curve consists of two turning points at pH 4.5 and 8.5, for which the authors provide a two‐site silica surface model with different acidity and the predominant presence of the less acidic sites (81 %). The origin of the two sites is proposed to stem from different hydrogen‐bonding environments: The more acidic silanol species are considered to interact with water directly, that is, they point towards the solution. The less acidic species are thought to interact with another silanol group, that is, lying in the surface plane. Elsewhere, these two species are also referred to as geminal and vicinal silanol groups, respectively.^[16d]^

Based on calculations of the surface potential using the Gouy–Chapman model, which assumes a low electrolyte concentration, the *χ* ^(3)^ term was deduced and used to infer the pH dependence of the surface potential at a high concentration where the Gouy–Chapman model is not strictly valid. The result suggested that the maximal surface potential for the silica–water interface with 0.5 m salt is 140 mV at pH 12.

In 2004, Shen and co‐workers performed the first pH‐dependent SFG study of the silica–water interface by measuring the O‐H stretch vibration under SA geometry with ssp polarization.^[19b]^ In all spectra, they observed two broad resonances at around 3200 cm^−1^ and 3400 cm^−1^ for the more and less strongly hydrogen‐bonded water. However, they realized that compared to α‐quartz, the low‐frequency band of fused silica appeared broader and shifted to slightly higher frequencies, thus suggesting that water at crystalline surfaces is more structured. After tuning the pH value between 1.5 and 11 without keeping the ionic strength constant, they observed an overall monotonic increase in the SFG signal with increasing pH value, which is in line with the previous SHG results by the Eisenthal group.^[14a]^ However, they observed that the intensity of the low‐frequency band varies with the pH value just like the SHG signal, while the high‐frequency feature hardly shows any pH‐sensitivity at all. In a time‐resolved study by the same group, it was found that around a neutral pH value, the vibrational lifetime of the O‐H stretching band is about 300 fs, that is, close to that of bulk water.^[25]^ Follow‐up studies by Borguet and co‐workers, as well with ssp polarization, demonstrated that the vibrational lifetime becomes longer as the surface charge decreases, which in the case of the silica–water interface corresponds to a decreasing pH value.^[26]^ Around pH 2, where the silica surface is neutral, and SFG is sensitive to the first few interfacial water layers, the authors observed lifetimes as high as 570 fs. By contrast, at a high surface charge (pH 12), where the SFG probing depth is, in principle, limited by the Debye length of the associated surface electric field, the vibrational dynamics are faster (ca. 255 fs) and are interpreted as bulk‐like.

In 2012 the Cremer group^[27]^ employed SFG to study the surface affinity of Hofmeister cations at the negatively charged silica surface at pH 10. Deviations from the usual Hofmeister series were observed for the Li^+^ ion, which were explained by its strong hydration in aqueous solution.

A whole series of pH‐dependent studies of the silica–water interface was performed by the Gibbs‐Davis group, starting from 2012.^[16a]^ These studies present pH scans with SHG under EW geometry with the s‐in/all‐out polarization combination. In this work, they studied the impact of the cation size on the pH‐dependence by using four different alkali salts at 0.5 m as background electrolytes. For all the alkali salts, they observed a bimodal titration curve, as presented by the Eisenthal group.^[14a]^ However, the inflection point for the high p*K*_a_ species varied substantially depending on the chosen salt (from 8.3 (NaCl) to 10.8 (LiCl)), which suggested that the stability of the less acidic silanol groups depends on the ion identity. These conclusions are drawn under the assumption that the change in the SHG signal can be directly correlated with deprotonation of the interface. Furthermore, the relative ratio of the two silanol species also seemed to depend on the salt: From the approximate 20:80 ratio for more/less acidic sites in an NaCl electrolyte proposed in the Eisenthal studies presented above, the relative abundance of the more acidic site can increase to 60 % by using LiCl. Since the ion‐specific surface acidity does not scale with the ionic radius but increases in the series Na^+^<K^+^<Cs^+^<Li^+^, they concluded that several effects contribute.

Cations may perturb the interfacial water structure and/or stabilize siloxide, which is why all interactions, that is, ion–surface, ion–water, and water–surface have to be considered:

1. Small, hard ions such as Cs^+^ interact more strongly with (i.e. stabilize) the hard siloxide.
2. More hydration leads to more acidic sites, which holds except for Na^+^.
3. Matching water affinities can lead to ion paring with a shared hydration shell, which means that Na^+^ and siloxide match better than Li^+^ and siloxide.

Subsequently, they studied the impact of different halide anions on the pH dependence by employing the same experimental conditions.^[16b]^ They observed that with increasing halide size:

1. The p*K*_a_ value of the more acidic silanol species shifts to a lower pH value, and that of the less acidic one shifts to a higher pH value.
2. The titration curve gets sharper, which suggests increasing positive cooperativity between the larger (less hydrated) halides and the cation and the surface: They concluded that large anions promote deprotonation of the more acidic species through acid–base coupling between silanol neighbors.
3. The fraction of acidic sites increases (from 20 % to 86 % for sodium halides and from 45 % to 91 % for potassium halides), which means an increase in the surface charge at a neutral pH value.
4. The effective acidity of the less acidic sites decreases, which suggests that a high surface charge makes it more difficult for the less acidic sites to deprotonate.

Additionally, they concluded that the less acidic silanol sites show negative cooperativity: deprotonation of one silanol group inhibits deprotonation of the next one.

A complementary SFG experiment^[16b]^ interrogated the pH dependence of the O‐H stretch vibration with 0.5 m NaI background electrolyte. Under a ppp polarization combination and EW geometry, the authors observed an intensity increase not only from a neutral to a high pH value but also from a neutral to a low pH value, which differs from all the SHG results reported for this system. They concluded that the cooperative structure between the surface, the cation, and the anion stabilizes one dissociated and the other protonated silanol species at a neutral pH value. This structure displaces more interfacial water molecules than the structures formed at low or high pH values, which gives rise to a minimum in the SFG signal. Without providing an interpretation, they noted a 30–40‐fold decrease in the SFG response from pure water to 0.5 m NaI, much higher than that reported by the Hore group for NaCl (in ssp)^[20]^ and opposite to their own findings^[16b]^ when employing SHG, which showed a 2.5‐fold increase.

The same group also studied the impact of salt concentration on the previously discussed cation‐ and anion‐specific effects by comparing 0.5 m with 0.1 m solutions using SHG under s‐in/all‐out polarization.^[16c]^ They observed that the cation‐specific effects essentially vanish upon dilution to 0.1 m, while the anion‐induced changes of the pH dependence remain almost unaffected by dilution. They concluded that the alkali chlorides, except NaCl, stabilize the less acidic silanol species in the protonated form, which stems from surface–water–electrolyte interactions. This cation‐specific interfacial distribution becomes more similar for different alkali chlorides at a lower concentration. The halide–surface structure, on the other hand, seems to be so stable in the case of large anions that it already forms at 0.1 m.

**Figure 6:**
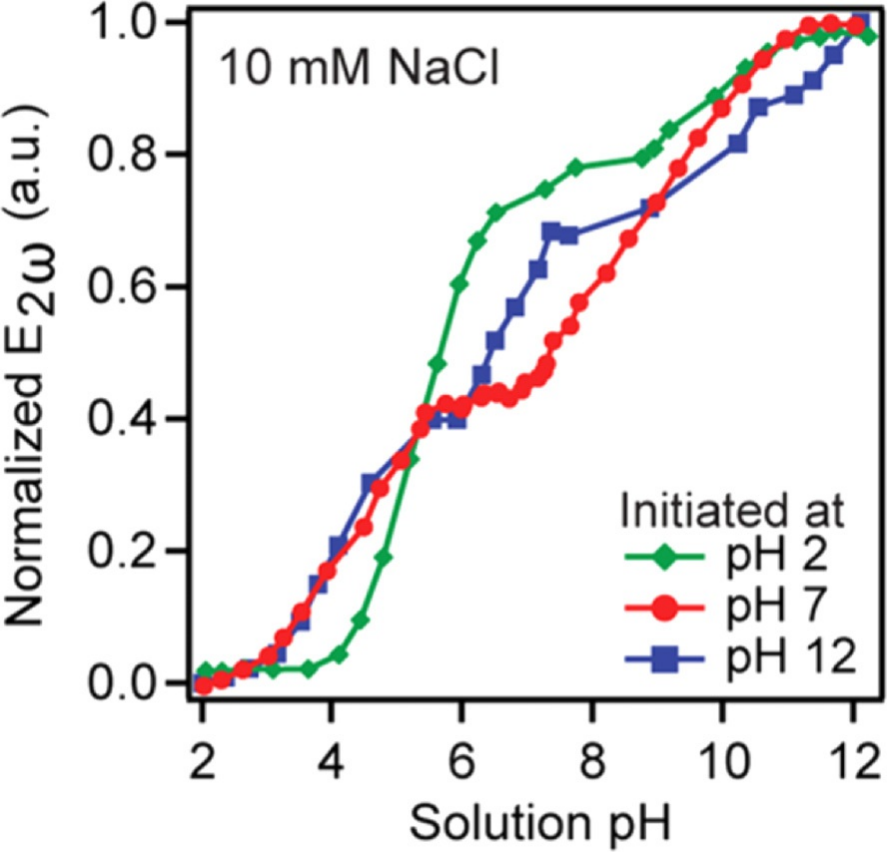
a) Normalized SHG intensity titration curves for an aqueous solution in contact with silica as a function of pH value. The titration was started at pH 2, pH 7, or pH 12, as indicated in the legend. Adapted from Ref. [16d] with permission. Copyright 2015 American Chemical Society.

Moreover, they reported a SHG study (p‐in/all‐out) that demonstrated that silica undergoes substantial hysteresis.^[16d]^ As Figure 6 illustrates, by titrating from different starting pH values, they observed that the titration curve may show two or three inflection points, thus indicating the presence of up to three differently acidic silanol sites with a changing relative abundance depending on the surface history. They further argued that the acidity is related to different hydrogen‐bonding environments of the protonated silanol with increasing acidity in the series:

1. isolated (hydrophobic) species,
2. geminal species that interact with water,
3. vicinal species that interact with neighboring silanols.

With 10 mm NaCl background electrolyte and using a p‐in/all‐out polarization combination, they deduced p*K*_a_ values of 3.8, 5.2, and ca. 9, which are all present if the starting pH value was 12, whereas from pH 7, species (1) and (3) were present, and from pH 2, species (2) and (3) were present. Recently, in a combined SFG and SHG study in collaboration with the Hore group,^[28]^ they showed that the SHG signal originates from the silica substrate and from the net order of water, with the substrate dominating at low pH values. The advantage of SFG in spectral resolution makes interpreting the SFG water signal corresponding to the net amount of ordered water difficult.

In 2013, Borguet and co‐workers published a study on the salt sensitivity of the SFG response from pH 2 to 12.^[17a]^ Under the ssp polarization combination and EW geometry, they found that adding 0.1 m NaCl to pure water led to the SFG response changing most dramatically around a neutral pH value, but being mostly insensitive to the addition of salt at low and high pH values: In the presence of a background salt concentration, they observed a monotonic increase in the SFG signal from pH 2 to pH 12. Without adding salt, however, they found a maximal response at around pH 8. The provided interpretation suggested that the interfacial water is most structured at a neutral pH value. However, in this study, the ionic strength of the pure water system was not kept constant across the investigated pH window, and varies between 10^−7^ for neutral and 10^−2^ for pH 2 or 12. Therefore, the Debye lengths of “pure water” and 0.1 m NaCl vary dramatically around a neutral pH value, but become comparable at low and high pH values.

In 2016, Chou and co‐workers presented an SFG study with the ssp polarization combination and using solutions of high ionic strengths (6 and 12) of different chloride solutions, namely NaCl, LiCl, MgCl_2_, and CaCl_2_ at a neutral pH value.^[14f]^ They found that divalent ions show a single low‐intensity, high‐frequency water band at around 3500 cm^−1^, which suggests that the interfacial water order is almost lost for these salts. They conclude that, in contrast to alkali ions, the local electric field of divalent ions is strong enough to polarize, reorient, or displace water interacting with the surface silanol groups.

In 2016, Tahara and co‐workers presented a phase‐resolved SFG study (ssp polarization) on the silica–water interface under neutral (pH 7.2), acidic (pH 2.1), and basic (pH 12.1) conditions.^[17b]^ They observed that at a neutral pH value and 10 mm background electrolyte, the low‐frequency part of the prominent double feature of the O‐H stretch disappears upon isotopic dilution. From this, they concluded that the 3200 cm^−1^ band, known to be the salt‐sensitive part (in ssp), is largely caused by intra‐ and/or intermolecular vibrational coupling. They further noticed that the uncoupled spectrum still varies in terms of its shape and intensity with the pH value, because of the positive/negative contributions at high/low frequency. The low‐frequency part appeared to be negative at a low pH value, positive at a high pH value, and with a negligible contribution at a neutral pH value. These contributions are interpreted as three different water species:

1. H‐up, bonded to the siloxide
2. H‐up, bonded to the silanol oxygen
3. H‐down, bonded to the DL water

According to their interpretation, tuning the pH value from basic to acidic increases the number of species (2) and (3) present at the interface. They noted that, in particular, the spectral component of species (3) shows a broad continuum of strong hydrogen bonds.

In 2017, the Gibbs group published two related SFG studies on the silica–water interface. The first one compared the pH dependence of the SFG response under the ssp versus pss polarization combination, as reproduced in Figure 5 b.^[17c]^ For pss, they observed a monotonic increase in the signal as the pH increased, very similar to what is known from SHG studies of the same system. Based on this finding, they concluded that pss is more surface‐sensitive and provides direct insight into the SL, which appears to be more ordered as the pH value increases. In contrast, the SFG intensity measured using ssp experiments shows a minimum signal at a neutral pH value and an increase when the conditions become more basic or acidic. They concluded that ssp provides a larger probing depth than pss and reports on the more outer water layers that, in contrast to the topmost waters, seem to flip around a neutral pH value. They rationalized the findings with two possible scenarios, both being in line with the net water flip observed with phase‐resolved SFG by Tahara and co‐workers.^[17b]^ One scenario considers a pH‐induced distortion of the SL hydration shells, the other considers the overcharging of the EDL at low pH values, which usually is only expected to occur for multivalent ions.

In the second study, they tested this non‐monotonic pH dependence of the ssp SFG response with respect to the cation species of a highly concentrated electrolyte solution (500 mm).^[17d]^ At a neutral pH value, which coincides with the minimum of the titration curve, they observed slight differences in the signal intensity in the series Cs^+^<K^+^<Na^+^<Li^+^, which they interpreted to arise from the surface propensities increasing from Li^+^<Na^+^<K^+^<Cs^+^. At low and high pH values, they observed inversion of this series, from which they conclude that the EDL model is only valid for a narrow range around a neutral pH value. For high pH values, they reason that the cation adsorption is mediated by hydration water, which may result in the expulsion of Cs^+^ ions but the specific adsorption of hydrated Li^+^ ions. The inversion at low pH values is attributed to a combination of EDL overcharging and asymmetric dehydration.

Recently, the Gibbs and Hore groups^[28]^ made a direct comparison between pH‐dependent SHG and SFG results by using various polarization combinations. They conclude that the silica substrate itself can significantly contribute to the SHG signal, especially at a low pH value. Moreover, as a result of the potentially spectrally separated, oppositely oriented water ensembles in SFG, care has to be taken when interpreting the SFG intensity.

### 3.3 The Free‐OH Debate on Silica

With an SA geometry, the SFG spectrum of the silica–water interface shows a broad band in the frequency region between 3200 and 3500 cm^−1^, commonly assigned to OH stretching vibrations of hydrogen‐bonded OH groups, as discussed above. However, recent work by the Tyrode group revealed the existence of an additional OH stretching band around 3680 cm^−1^, a frequency region often referred to as the free‐OH signature and indicating the weak intermolecular interactions of the associated OH species. This study, performed under ssp, ppp, and sps polarization, demonstrated that this high‐frequency band clearly appears after heating for 4 hours at 1000 °C when employing the EW geometry, which amplifies the high‐frequency region.^[29]^ A sharp band at about 3680 cm^−1^ is observed, which could be the signature of the OH stretch vibration of either isolated water molecules or isolated surface silanol groups. They additionally observed that this band is the predominant one for a silica–air interface, a nominally dry interface. However, for silica–air, the band seems to be blue‐shifted by about 70 cm^−1^ compared to its silica–water analogue. In addition, the authors performed experiments under different pH conditions, for different polarization combinations, and in the presence of a positively charged surfactant (CTAB) to deduce the relative signs of the corresponding bands. Overall, they concluded that the free‐OH band could be assigned to isolated silanol groups, which pointed away from the surface into the water.

Another study by Backus and co‐workers tested the validity of this interpretation by determining the phase information on the free‐OH band directly^[30]^ by employing phase‐resolved SFG with ssp. The authors found the bands of free‐OH and the OH stretch of hydrogen‐bonded OH groups to have the same sign, which indicates that the free‐OH group is oriented with the hydrogen atom pointing to the silica surface. Based on this finding, they interpreted the free‐OH vibration as arising from a weakly interacting water species rather than an isolated silanol species. This experimental finding was rationalized with MD simulations, which identified hydrophobic patches on the nominally hydrophilic surface as a consequence of the siloxane bridges being present at the silica surface. Together with this concept, the experimentally determined increased contact angle for preheated silica was thus interpreted as an increased number of hydrophobic patches on the surface, rather than an increased number of isolated silanol groups.

### 3.4 Summary of the Silica–Water Interface

The previous studies demonstrate that the nonlinear response of silica–water is sensitive to changes in the interfacial charge distribution. By adjusting the experimental conditions, it has thus been used as a reporter of both the effective surface charge as well as the local concentration of ions that screen these charges. The effective surface charge can be tuned by changing the pH value or, as has very recently been demonstrated, by changing the temperature.^[31]^ Moreover, the nonlinear response is also dependent on the surface preparation^[29]^ and most likely on the type of silica as well.

The SFG intensity of the O‐H stretch vibration of water in contact with a silica surface has been shown to consist of the typical double band, with maxima at around 3200 and 3400 cm^−1^,[^14b^, ^14c^, ^14f^, ^17a^, ^17c^, ^17d^, ^19b^, ^20^] that is also known for bulk water^[32]^ and the air–water interface.^[33]^ As indicated by isotopic dilution experiments, the low‐frequency part of this band is substantially affected by vibrational coupling.[^14g^, ^17b^]

Based on concentration‐dependent studies, the following consistent conclusions have been drawn:

1. In general, the nonlinear response increases as the salt content of the solution decreases,[^14a^, ^14b^, ^14d^, ^14e^, ^14g^, ^15^, ^16e^, ^20^] which can be assigned to a concentration‐dependent decaying length of the surface potential and qualitatively understood with the Gouy–Chapman theory of interfaces.[^14a^, ^14d^, ^14e^, ^14g^, ^15^, ^16e^, ^20^] However, a recent theoretical study claims that a good fit of SHG data with the Gouy–Chapman theory does not mean that the underlying physical situation corresponds to this model.^[34]^ Moreover, the resulting parameters might not have physical significance.
2. The concentration‐dependence of the nonlinear response seems not only to be affected by the ion valence but also its size.[^14b^, ^14e^]
3. At very low salt content (sub‐mm), the nonlinear response has an inverse concentration‐dependence, namely, a decreasing signal as the concentration is further decreased. This has been assigned to optical interference, which contributes to the signal when probing depths larger than tens of nanometers.[^14e^, ^15^, ^16e^]
4. The relative contribution of layers close to the surface to the total nonlinear response is increased under the EW geometry, compared to the SA geometry. This is evidenced by comparably high signals at high ionic strength, and the impact of interference shifted to a lower concentration.[^14e^, ^15^, ^20^]
5. The low‐frequency part of the O‐H stretch SFG band is more sensitive to the variation of salt concentration and thus preferentially results from the field‐induced contribution *χ* ^(3)^,[^14a^, ^14d^, ^14e^, ^14g^, ^20^] which reports on the more distant water layers,[^14d^, ^14e^, ^14g^, ^20^] in agreement with the reported bulk *χ* ^(3)^ for water underneath a monolayer of charged lignoceric acid.^[21]^ At a high salt concentration (*c* ≥0.1 m), the spectral weight of the band shifts to higher frequencies, independent of the salt species,[^14b^, ^14e^] and is less affected by vibrational coupling.^[14g]^

The following findings are based on the pH‐dependent studies:

1. The nonlinear response of the silica–water interface is lowest at pH 2 and increases monotonically upon increasing the pH value, which is interpreted as a corresponding increase in the surface charge.[^14a^, ^16a^, ^16b^, ^16c^, ^16d^, ^19b^]
2. Depending on the cation and anion species, the concentration, and the titration direction, the titration curve shows two or three inflection points at different pH values. This is interpreted as reflecting the presence and ratio of different types of surface silanol groups that contribute, together with the acidity, to the overall surface charge.[^14a^, ^16a^, ^16b^, ^16c^, ^16d^] More precisely, it was concluded that there are isolated, geminal, and vicinal species with p*K*_a_ values that increase in this order.^[16d]^
3. The SFG studies provided further insight and have demonstrated that the interfacial water structure is more complex than assumed based on the SHG results.[^14f^, ^16b^, ^17^, ^19b^, ^35^] In contrast to SHG, the pH‐dependent trend of the (ssp) SFG response is not monotonic in the presence of salt but shows a minimum around neutral to slightly acidic pH values.[^17c^, ^17d^, ^35^] This suggests a net water flip in this pH range, which was supported by phase‐resolved SFG results.^[17b]^ Possible scenarios giving rise to this flip are a pH‐induced distortion of SL hydration shells, EDL overcharging,[^17c^, ^17d^, ^35^] and different types of water present at the surface.^[17b]^
4. The SFG response in the pss polarization combination shows a monotonic trend,^[17c]^ similar to what is observed in SHG studies with the analogous polarization combination, s‐in/all‐out.[^16a^, ^16b^, ^16c^, ^16d^] In the pss polarization combination, the spectral weight of the O‐H stretch of the silica–water interface is more on the high‐frequency band compared to ssp.^[17c]^ Together with the pH‐dependent trends, it was concluded that the two polarization combinations are sensitive to different types of water, probably at different distances from the surface.[^17c^, ^17d^, ^35^]

## 4 Alumina

Another important, ubiquitous mineral whose surface charge is determined by deprotonation of surface hydroxy groups is alumina. Sapphire and corundum are the natural forms of alumina with trace amounts of impurities. This oxide serves as another good model system for studying differently charged aqueous interfaces. The difference compared to silica is, however, that these hydroxy groups can be deprotonated (negatively charged), protonated (neutral), as well as over‐protonated (positively charged) by tuning the pH value accordingly.

By employing SFG with EW geometry and ppp polarization, Pink and co‐workers studied the pH dependence of the sapphire–water interface and found a minimum in the titration curve around pH 8.^[36]^ Changing the pH value to acidic or alkaline conditions led to an overall increase in the OH‐stretch absorption band, which is composed of three resonances. By comparing the spectra recorded at low and high pH values, the authors further observed substantial differences in the spectral shape. This difference was rationalized by a change in the sign of individual band features, which indicates a net 180° flip of the associated water molecules. They concluded that the SFG minimum determined at pH 8 was the isoelectric point. Additionally, by comparing hydrated with dehydrated sapphire surfaces they demonstrated that the overall O‐H stretching band scales with the number of surface hydroxy groups.

In 2001, Eggleston and co‐workers performed SHG measurements on the corundum–water interface under the EW geometry.^[37]^ With a background electrolyte concentration of different sodium salts between 1 and 100 mm, they studied the pH‐dependence of the SHG response. In the titration curves, they found an inflection point around pH 5–6, which matched with the point of zero salt effect and was, therefore, interpreted as the pzc. Compared to commonly accepted pzc values for alumina powders (ca. pH 8–9.4) and the previous SFG study without additional salt, this value was surprisingly low. Furthermore, they found acceptable agreement between the dependence of the SHG signal on the ionic strength and the Gouy–Chapman model for the screening of surface charge.

In 2005, Eisenthal and co‐workers performed SHG experiments on the same system using the SA geometry with p‐in/p‐out polarized light.^[38]^ After adding 1–100 mm NaNO_3_ as the background electrolyte, they compared the pH dependence of different faces of corundum, namely the (0001) and (1$\bar{1}$ 02) surfaces. They found that the pzc of single‐crystalline alumina is not only significantly more acidic than those of alumina powders, but the acidity also depends critically on the crystal face, with pH_pzc_ 4.1±0.4 for (0001) and pH_pzc_ 5.2±0.4 for (1$\bar{1}$ 02). This difference in surface acidity was assigned to differences in the coordination environment and local structure of associated hydroxy groups.

**Figure 7:**
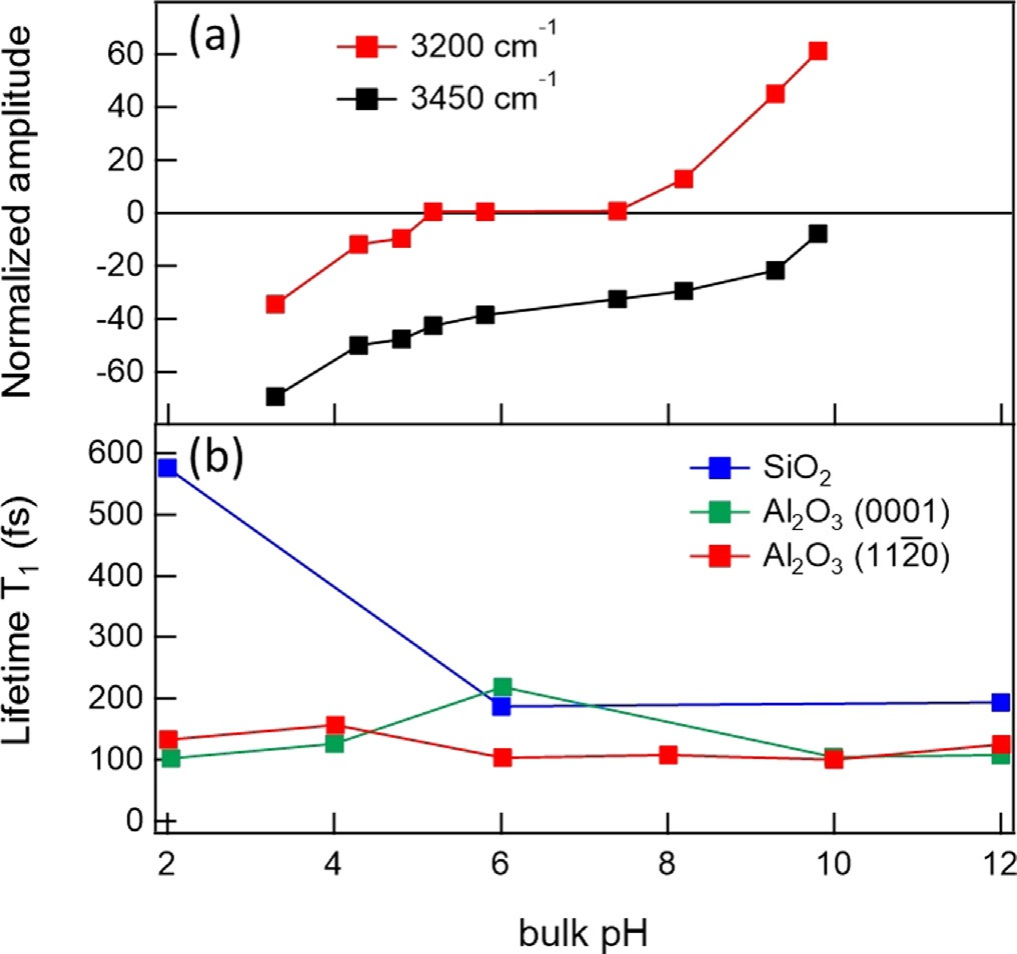
a) Amplitude of the 3200 and 3450 cm^−1^ signals obtained from fitting the SFG intensity spectra as a function of the pH value at the Al_2_O_3_(0001)‐H_2_O interface. Adapted from Ref. [39] with permission. Copyright 2017 American Chemical Society. b) Vibrational lifetime of the interfacial OH species at the SiO_2_–H_2_O interface (blue), the Al_2_O_3_(0001)–H_2_O interface (green), and the Al_2_O_3_(11$\bar{2}$ 0)–H_2_O interface (red). Adapted from Ref. [40] with permission. Copyright 2017 American Chemical Society.

In 2008, Shen and co‐workers reported an SFG study (in SA geometry) on the pH dependence of the (0001) surface and amorphous alumina^[39]^ with and without the addition of 0.1 m NaCl as a background electrolyte and using different polarization combinations. In the spectra, they observed the typical double feature in the O‐H stretch region of hydrogen‐bonded OH groups, with signals at 3200 and 3450 cm^−1^ and an additional band in the free‐OH region, which is in line with previous SFG studies.^[36]^ The “free‐OH” band was interpreted as a reporter of the surface hydroxy groups, for which an average tilt angle of about 26° was determined based on the polarization dependence of this band. For the H‐bonded O‐H stretch band, they found substantial differences in the spectral shape upon variation of the bulk pH value between acidic and alkaline conditions. The results of the spectral analysis are reported in Figure 7 a, which shows that the band at 3200 cm^−1^ flips sign around pH 6, while the band at 3450 cm^−1^ stays negative. Phase‐resolved data confirm the flip of the net water orientation for the ensemble resonating at 3200 cm^−1^. They concluded that the flip in the water orientation reports on the surface charge upon variation of the pH value as a result of the protonation/deprotonation state of surface hydroxy groups. For alumina (0001), the pzc value was determined to be around pH 6.3, which is again significantly lower than what is known for alumina powder or what they observed for amorphous alumina (ca. pH 8). This was interpreted as different forms of Al_*n*_OH species existing on these surfaces. Additionally, they found that, if the pH value is far from the pzc value, the O‐H stretch band of the hydrogen‐bonded OH groups decreases substantially upon the addition of 50 mm NaCl, which was interpreted as a screening of the surface charge. In the same year, another study^[41]^ reported similar SFG spectra (ssp and ppp) and pH dependence for the corundum (001) surface, with the data fitted with many resonances by assuming many different interfacial OH species. Also in 2008, Braunschweig et al.^[42]^ showed that surface disorder on a nanometer scale has a fundamental influence on the molecular structure at the (0001) interface. They observed no SFG band in ssp above 3600 cm^−1^ for an annealed surface with atomically flat terraces. For an unannealed sample with a higher roughness, a band between 3630 and 3680 cm^−1^ appeared, which is in line with, for example, the Shen study from 2008.^[39]^ The authors conclude that this high‐frequency band originates from aluminol groups in nanopores of the disordered surface. Moreover, they report a different pH dependence on the flat and rough surface, which indicates that the p*K* value for the deprotonation of aluminol groups at defect sites is different from that of atomically smooth terraces.

**Figure 8:**
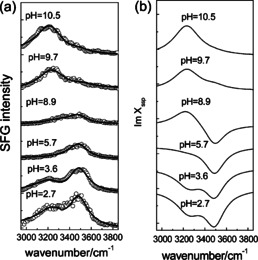
a) Intensity and b) Imχ^(2)^ spectra in the O‐H stretching region for the α‐Al_2_O_3_(1$\bar{1}$ 02)–water interface at different pH values. Adapted from Ref. [43] with permission. Copyright 2011 American Chemical Society.

The spectrum for the (1$\bar{1}$ 02) interface in contact with water is different from that of the (0001) surface: only two bands at 3230 and 3490 cm^−1^ have been reported.^[43]^ The lower frequency band (Figure 8) is assigned to interfacial water molecules, whereas the higher frequency band originates from the hydrogen‐bonded hydroxy groups of AlOH_2_ on the surface. From the sign reversal of the 3230 cm^−1^ band between pH 5.7 and 7.8, the authors concluded that the pzc value is about 6.7. The p*K* value for the deprotonation of AlOH_2_ is around 9.5. For the (11$\bar{2}$ 0) interface in contact with water, the frequency of the dangling OH group of Al_2_OH has been reported to be similar to that of the (0001) interface.^[44]^ A free induction decay study^[45]^ on the (1120) surface provides indications that this free‐OH stretch mode might consist of two modes centered at 3644, assigned to aluminum hydroxy groups, and 3679 cm^−1^, attributed to a free‐OH stretch of interfacial water, with dephasing times of about 90 and 900 fs, respectively. The SFG response of the interfacial water molecules at about 3200 and 3400 cm^−1^ at this (11$\bar{2}$ 0) interface is sensitive to the pH value, with a minimum in the SFG intensity around pH 6.7.^[44]^ The 2016 results of the Borguet group^[46]^ are in line with these observations. However, they additionally observed a signal at about 3000 cm^−1^, which was particularly clear with ppp polarization and became even more pronounced in the presence of ions. The authors assign this band to chemisorbed surface OH groups (i.e. aluminol groups) strongly hydrogen‐bonded to the surrounding OH groups and/or to interfacial water molecules that form strong hydrogen bonds with the surface aluminol groups. IR pump/SFG probe experiments show that these OH groups undergo very fast vibrational relaxation independent of the pH value of the aqueous solution, and thus the surface charge, as well as the ionic strength. The presence of the very strongly hydrogen‐bonded species that resonated at 3000 cm^−1^ could explain the very fast vibrational relaxation. In an ambient atmosphere, only the hydroxy signals appear in the spectrum for the (11$\bar{2}$ 0) surface.^[44]^ The Campen group showed that this band at about 3700 cm^−1^ also appears in the ssp SFG spectrum for a hydroxylated (0001) surface in ambient air.^[47]^

In 2017, Borguet and co‐workers presented a time‐resolved ppp SFG study of alumina with different exposed crystal facets, pH conditions, and background electrolytes.^[40]^ For the static spectra, they observed a blue‐shift in the H‐bonded O‐H stretching band for water at Al_2_O_3_ (0001) compared to Al_2_O_3_ (11$\bar{2}$ 0), which was interpreted as a comparably weak hydrogen‐bonding network. In line with this conclusion, they found, as shown in Figure 5 b at pH 6, where the surface is more or less neutral, a factor of two slower dynamics of that band for water at the (0001) face, compared to the (11$\bar{2}$ 0) face. At a charged alumina (0001) face, they observed faster dynamics for this band than what is known for bulk water or water at charged silica surfaces (Figure 7 b). This was interpreted to result from a) a fast proton transfer and/or b) efficient coupling of the O‐H stretch band with the bending overtone. In contrast to what is known for silica, they found no effect on the dynamics upon the addition of salt (0.1 m NaCl). In 2018 they showed that the addition of 0.1 m NaF led to the vibrational relaxation of water next to a positively charged alumina surface slowing down by a factor 4, which suggests that F^−^ alters the interfacial hydrogen‐bonding environment.^[48]^ The shielding effect of the halide ions on the SFG intensity next to a positively charged alumina surface followed the direct Hofmeister series, with minor exceptions. At the negatively charged surface, an anion‐specific effect following the indirect Hofmeister series has been observed, possibly originating from formation of an ion pair with the Na^+^ ion. Very recently, they showed that monovalent cations have a lower binding affinity than divalent cations to the (0001) surface.^[49]^ Moreover, the monovalent ions only attenuate the SFG signal, whereas the divalent ones increase the spectral intensity in the 3400 cm^−1^ region compared to that in neat H_2_O at pH 10. Time‐resolved experiments show that the cation‐induced restructuring of the water layer does not influence the lifetime of the vibrational energy redistribution.^[49]^

A recent review by Lützenkirchen et al. highlights the dependence of sample preparation on the isoelectric point of sapphire.^[50]^ As such, care has to be taken when comparing data from differently prepared samples.

## 5 Calcium Fluoride

### 5.1 pH Dependence

Another transparent mineral whose surface charge can be tuned by varying the pH value is calcium fluoride. In 2001, Becraft and Richmond studied the pH dependence of the O‐H stretch spectrum.^[51]^ They observed a large, broad SFG signal in ssp at a low pH value which they assigned to water strongly oriented by the positive charge of the CaF_2_ generated by the dissolution of fluoride ions. Upon approaching a neutral pH value, the signal intensity decreased as a result of the reduction of the surface charge. At a high pH value, a narrow signal at 3657 cm^−1^ has been observed that originates from Ca‐OH groups generated by ion exchange of F^−^ and OH^−^. In a phase‐resolved experimental and theoretical SFG study from 2016, Sulpizi and co‐workers^[52]^ showed that the H atom of the O‐H oscillators point toward the surface at low pH values, thereby proving the positive surface charge proposed by Becraft and Richmond. Moreover, a comparison with simulated spectra shows that the surface charge originates from fluoride defects rather than from proton addition to the surface, as proposed as a potential mechanism for the positive charge in the literature.^[53]^ Recently, the Backus and Sulpizi groups^[54]^ showed in a joint experimental and theoretical time‐resolved and 2D‐SFG study (ssp) that the localized charge defects pin water molecules at the interface, thereby resulting in very fast spectral diffusion and vibrational relaxation. At high pH values, the OH group was shown to point into the bulk, as expected for a Ca‐OH band. This is in clear contrast with a similar type of signal observed for the silica interface, where this signal originates from water pointing to nanoscale hydrophobic patches on the SiO_2_ (see Section 2.3). A ppp study with free induction decay by the Borguet group,^[55]^ with timescales of 70 and 50 fs for the strong and weakly hydrogen‐bonded water ensembles, revealed the presence of two oppositely oriented water populations at 3140 and 3410 cm^−1^ at a neutral pH value, which is in line with the phase‐resolved spectrum in Ref. [52]. In the case of D_2_O at pD 3.7, a roughly twofold slower free induction decay has been reported using ssp polarization.^[56]^ Moreover, they concluded that the hydrogen‐bonding network of water is dynamically heterogeneous, as different dynamics of the vibrational coherence were observed for different sub‐ensembles of hydrogen‐bonded water molecules.

### 5.2 Counterion Dependence

In a follow‐up study of their early work, Richmond and co‐workers studied, at a fixed positive surface charge (pH 5.8), the ionic strength dependence of the O‐H stretch SFG response of the CaF_2_–water interface for different salt solutions between 10^−5^ and 0.1 m.^[57]^ With the EW geometry, they observed that the SFG signal decreases with increasing salt concentration for all the salts, which is in line with the notion of surface charge screening in Gouy–Chapman theory. However, they found that the signal is more sensitive to the addition of SO_4_^2−^ and F^−^ salts than to Cl^−^ and Br^−^ salts. They concluded that sulfate and fluoride screen the surface charge more efficiently than chloride and bromide. They also observed substantial spectral deviation from the pure water spectrum in the case of fluoride, which they interpreted as additional disruption of the interfacial water structure.

**Figure 9:**
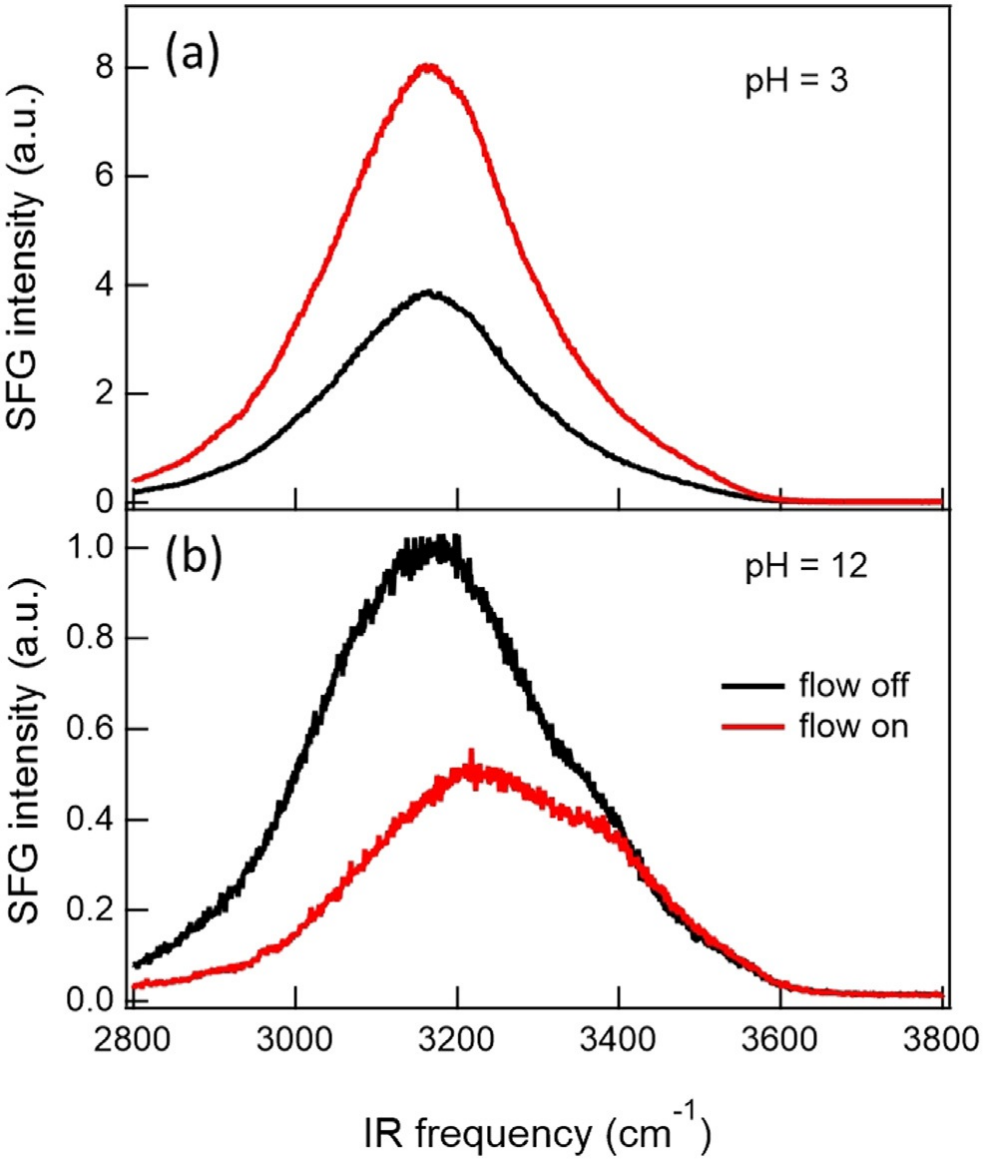
SFG spectrum (not normalized for the spectral envelope of the IR pulse) of the OH stretch region of the CaF_2_–water interface at a) pH 3 and b) pH 12 under static (black) and flow (red) conditions. Adapted from Ref. [5] with permission from AAAS.

In 2014, Bonn and co‐workers published a study on the impact of flow on the SFG spectra of water at the CaF_2_ surface.^[5]^ By using the EW geometry and ssp polarization combination, they compared flowing and resting water at different pH values. Under resting conditions, the highest response was observed at a low pH value, which was assigned to the interfacial order imposed by the positively charged surface as a consequence of preferential F^−^ dissolution. Around pH 8, a minimum SFG signal was observed, followed by an increase under alkaline conditions, which was interpreted as the result of dissolved carbonate replacing surface fluoride, which makes the surface more negative. Under flow conditions, as illustrated in Figure 9, the authors observed an increase in the signal at a low pH value and a decrease at a high pH value. At pH 9.5, flow changes the absolute intensity only slightly, but alters the spectral shape, which indicates a change in the sign of the band and a net flip of interfacial water molecules. These results were interpreted as a flow‐induced modification of the surface charge, which, compared to static conditions, requires a change in the pH value of up to 2 units. Similar flow‐dependent changes at low pH values are observed with SHG in p‐in/p‐out and p‐ and s‐in/s‐out polarization.^[58]^

## 6 Titanium Dioxide

The interaction of TiO_2_ surfaces with water is of great interest owing to the photocatalytic activity of TiO_2_. One of the first nonlinear spectroscopic studies of this interface was presented by Cremer and co‐workers in 2004.^[59]^ With 30 mm of different background electrolytes, they investigated the pH dependence of the OH stretch SFG (ssp) response at thin films of TiO_2_ (0.9–3.9 nm) on a silica substrate. Upon addition of NaCl, they found a minimum in the double‐featured band at around pH 4–6, which matched with the isoelectric point commonly known for this system. Above and below that pH range, they observed an increase in the low‐frequency feature (ca. 3200 cm^−1^) relative to the high‐frequency one (ca. 3400 cm^−1^). Adding PBS (phosphate‐buffered saline) buffer instead of NaCl shifted the minimum to pH 2, which was interpreted as a shifted isoelectric point because of the strong adsorption of phosphate on the TiO_2_ surface, similar to what they observed for silica. In the same year, Nihonyanagi and co‐workers studied the adsorption behavior of water from the vapor phase on TiO_2_ with and without pretreatment of the TiO_2_ surface by UV irradiation.^[60]^ Under EW geometry and the ppp polarization combination, they found that UV irradiation of the surface increases the intensity of the overall H‐bonded OH stretch as well as of a third feature in the free‐OH stretching region. They concluded that UV irradiation leads to more ordered water on the surface as a result of the increased hydrophilicity of TiO_2_. In 2012, the Cremer group published a follow‐up study in which they determined the effects of cations on the interfacial water structure at the negatively charged surface.^[27]^ It was observed that the cations followed, in principle, the Hofmeister series with a few exceptions, potentially arising from electronic properties, charge density, and hydrogen‐bonding ability.

**Figure 10:**
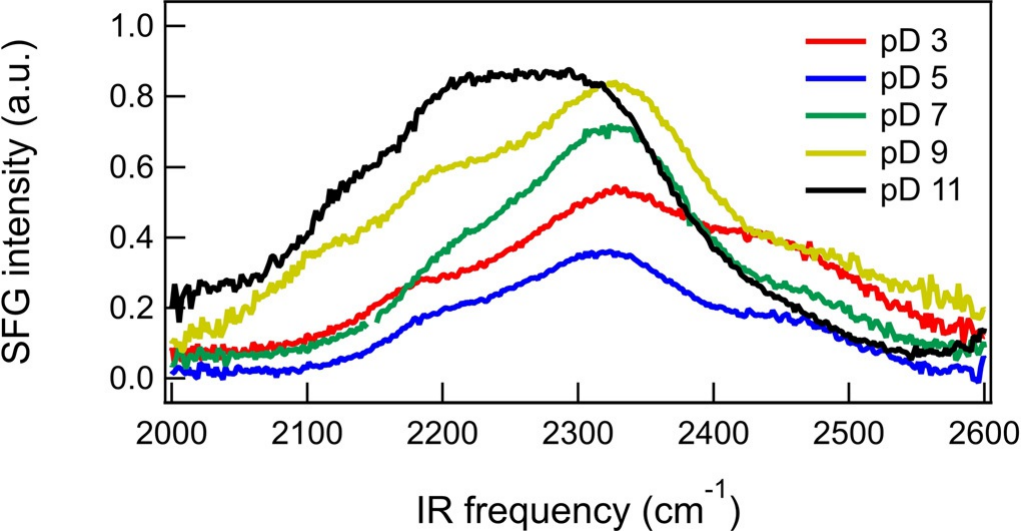
SFG intensity spectra in the O‐D stretch region of the TiO_2_–D_2_O interface at different pD values for a TiO_2_ layer with a thickness of 150 nm. From Ref. [62]—Published by the PCCP Owner Societies.

In 2017, Backus and co‐workers presented an ssp SFG study of water at UV‐irradiated thin films of anatase TiO_2_ (1 μm, consisting of 50–200 nm globular particles) deposited on a CaF_2_ substrate.^[61]^ They found that the two bands of the H‐bonded OH stretch have opposite signs, which indicates two sub‐ensembles of OH groups at the surface. The high‐frequency band was interpreted as representing weakly hydrogen‐bonded, chemisorbed OH groups at the surface that point towards the bulk water. The low‐frequency band, which indicates strong hydrogen bonding, was interpreted as physisorbed water that interacts with the chemisorbed species by pointing towards the surface. The superhydrophilicity of the UV‐irradiated surface was assigned to the strong interaction between the chemisorbed and physisorbed water species. Additionally, they observed significant changes in the double‐band shape upon isotopic dilution, which was assigned to vibrational coupling. In a different study, they examined the pD dependence of the OD‐stretch SFG response at 85 and 150 nm thin amorphous films of TiO_2_, as depicted for the 150 nm thick film in Figure 10, ^[62]^ They observed a minimum in the titration curve at around pD 5 which originates from a change in the sign of the main band at around 2300 cm^−1^ when the pD crosses the pzc. This main band originates from water in the first two layers. Additionally, high‐ and low‐frequency bands are observed that do not change sign as a function of pD and are assigned to TiOD groups and D_2_O molecules, respectively, directly hydrogen bonding with the surface.

## 7 Mica

Since mica is one of the most abundant minerals on Earth, it has also been gaining increasing attention, especially in regard to its interactions with water. In 1998, Salmeron and co‐workers presented the first SFG study of the mica–water interface, using ssp polarization, where they investigated the adsorption behavior of D_2_O depending on the humidity.^[63]^ They observed that the typical double band appeared in the D‐bonded OD‐stretching region as the humidity was increased, with the low‐frequency feature becoming the predominant signal. They concluded that, as the humidity increases, the sub‐monolayer water structure evolves into a more ordered D‐bonding network with the complete absence of the free‐OD peak at full monolayer coverage. Another SFG study employing the SA geometry demonstrated an azimuthal angle dependence of the OH‐stretch response at mica (001), which indicated the mica crystal structure imposes anisotropy on the interfacial water response.^[64]^ As discussed in more detail in Section 8.2.2, several groups^[65]^ have studied the mica–water interface in the context of ice nucleation, concluding that the surface‐induced ordering of water plays an important role in the freezing process of water.

## 8 Outlook

### 8.1 Experimental Challenges

As mentioned before, nonlinear spectroscopy is not only surface‐sensitive but also EDL‐selective due to its symmetry selection rules. Intrinsically, nonlinear spectroscopy provides information about all the non‐bulk‐like interfacial water layers which lack centrosymmetry. Therefore, it reports not only on the surface charge but also on the distribution of the counter charges that screen the surface and form the EDL. In other words, the degree to which each individual water layer contributes to the nonlinear (NL) signal is primarily determined by the decay of the potential associated with the surface charge. However, this convolution of signal contributions from water layers close to the surface (*χ* ^(2)^) as well as distant water layers (*χ* ^(3)^) makes the analysis complicated, as has been discussed in this Review, since any change in the EDL will affect the overall nonlinear response. For that reason, the experimental conditions of both the interface and the optical setup are crucial when interpreting the results and meaningfully comparing different studies.

A recurrent issue in many second‐order nonlinear spectroscopic studies of water at charged interfaces is the fact that symmetry breaking may result not only from the reorientation but also from the polarization of interfacial water molecules. So far, it has not been possible to disentangle the two contributions to the NL signal properly. Only water species that differ in their hydrogen‐bonding strength and/or net orientation can be discriminated using SFG (e.g. Refs. [14a, 14c, 17c, 19b]) and phase‐resolved SFG (e.g. Refs. [14g, 17b, 19b]). However, the hydrogen‐bonded OH stretch is a broad multifeatured band that may still hide chemically similar sub‐ensembles of polarized and/or reoriented water species. Although phase‐resolved SFG is evidentially a powerful tool, we caution the reader about overinterpreting low‐intensity phase‐resolved spectral features, as this can easily result in misleading conclusions. A good example is the assignment of the low‐frequency part of the OH stretch in the water–air spectrum, which has been the subject of intensive discussion,^[66]^ and seems to be a normalization artifact.^[67]^ Even if artifacts can be excluded, the band shape might not only report on the distributions of water ensembles under different chemical environments but could also be affected by vibrational coupling, as demonstrated for the silica–water interface as well.[^14g^, ^17b^] If this is the case, isotopically diluted water can be used to suppress the coupling effects and afford the “absolute” band shapes.

Another experimental option is to measure the NL response in an off‐resonant fashion by employing SHG. It has been argued that this method provides a more direct evaluation of the surface charge, as the signal seems to be less affected by orientational effects.^[17d]^ However, it is still unclear how SHG then reflects the balance between reoriented and polarized layers if not in an additive fashion. Additionally, the chemical selectivity of the NL response is lost in SHG, and it is not known how the SHG signal depends on the probing wavelength. All of this should be taken into account when comparing SHG with SFG studies and also of different SHG studies.

Moreover, for both methods, SFG and SHG, the employed beam geometry is also important for interpreting the NL signal. Since a variation in the beam angle is known to alter the local field at the interface, it may affect both the shape and absolute intensity of the spectrum. A recent study on the TiO_2_–water interface serves as a good example of this problem.^[62]^ In principle, this effect can be corrected for by invoking Fresnel factors,^[68]^ but this requires accurate determination of the refractive indices of the involved media and is, therefore, rarely done on these systems. An often‐used geometric trick to amplify the NL response is to tune the incident beam angles to total internal reflection, which generates an evanescent field at the interface. This is especially beneficial for kinetic experiments, as it allows the accumulation time to be reduced by roughly a factor 100.[^5^, ^69^] On the other hand, the EW geometry results in the NL response being more surface‐sensitive, which distorts the signal contributions from layers close and distant from the surface in favor of the closer ones.^[18]^ Depending on the interfacial charge distribution, this might even lead to a loss of the EDL selectivity if the Debye length exceeds the EW.

The minerals that can be studied by NL spectroscopy have to be transparent in the frequency ranges of all the involved beams. For highly absorptive materials, thin films deposited on a transparent material could be used. The preparation of well‐defined thin films is experimentally challenging, and the measurement can be affected by multiple reflections, fluorescence, or other unknown contributions to the nonresonant background.^[62]^ However, even for the most extensively studied interface, silica–water, there is no unifying picture for the nature of the EDL. Historically, Stern (SL) and diffuse layer (DL) have been approached separately through a) pH titration with a high electrolyte background concentration and b) tuning the ionic strength around a neutral pH value. Whereas (a) is sensitive to changes in the surface charge, (b) primarily reports on the Debye length by going even to sub‐mm concentrations. In this context, the pretreatment of the surface with respect to both the pH value and ionic strength is crucial for determining its actual state, since silica seems to undergo massive hysteresis during pH titration^[16d]^ and ion exposure.^[15]^ In addition, the above‐mentioned approaches come with a few caveats:

1. At a high salt concentration, the dissolved ions themselves may affect the surface charge by promoting protonation or deprotonation. Hence, the surface potential and the zeta potential are related to one another and cannot be treated separately.
2. Although the Gouy–Chapman model has been shown to provide a good qualitative prediction of the ionic strength dependence of the NL response,^[14e]^ it does not yield a satisfying description over the entire concentration range. For the model to work, dramatic changes in the screening properties need to be invoked by, for example, changes in the interfacial dielectric permittivity.[^15^, ^24^] This implies that some information is contained in the NL response that has so far not been considered. Therefore, we caution the reader about relying on quantitative arguments made from GC‐based model descriptions of these types of SFG and SHG experiments.

In addition, strong indications exist that SHG and SFG report differently on the nature of EDLs (e.g. Ref. [16a] versus Ref. [17d] and Refs. [15, 24] versus Ref. [14e]). Primarily, this difference is ascribed to orientational effects, which are assumed to add up differently in SHG and SFG.^[17c]^ How exactly these effects come into play and why they differ for the two technical options are interesting as well as being crucial issues that have to be addressed in the future. Otherwise, quantitative conclusions about the electrochemical properties of the studied interfaces are meaningless, if not completely misleading. For future studies, a comprehensive study that unifies the EDL picture independently of the technical approach would be a desirable goal.

All in all, nonlinear spectroscopy has provided new fundamental insights into mineral–water interfaces beyond the Gouy–Chapman description of electric double layers. To highlight a few examples: It has been demonstrated for the silica–water interface that not only oppositely oriented sub‐ensembles of water,[^14g^, ^17b^] but also hydrophobic species exist at this nominally hydrophilic surface.^[30]^ Furthermore, several species of surface hydroxy groups with greatly different acidities have been identified.^[14a]^ The screening properties of monovalent salts have been used to relate the NL response to interfacial ionic strengths and monitor interfacial dissolution kinetics.[^14e^, ^69b^] Another interesting and persistent question related to those studies is how the screening length of the surface potential and the surface charge are correlated. To approach this problem, one could think of using an electrode to apply an external potential. However, this electrode material would still have to fulfill the optical constraints for SFG/SHG. The first attempts in this direction have been made using graphene as the electrode material deposited on a transparent substrate,^[70]^ but the chemical challenges that come along with this approach will still require more engineering efforts in the future.

### 8.2 Potential Consequences for Geochemistry

#### 8.2.1 Mineral Dissolution

As discussed in Sections 3–7, extensive studies employing nonlinear spectroscopic techniques have provided in‐depth insights into the electrochemical and acid–base properties of various mineral–water interfaces. In nature, the contact between minerals and water occurs on a variety of timescales, thus making kinetic observations relevant for geochemistry. However, only a few studies have started to focus on the kinetic behavior of these interfaces, which ultimately determines the chemistry of these kinds of systems. In 2008, Geiger and co‐workers reported an SHG study with the EW geometry and p‐in/all‐out polarization in which they temporally resolved the pH titration of the silica–water interface in the presence of 10–500 mm salt.^[69a]^ They found that the surface lags spatially and temporally behind the bulk pH value, which, in the case of the temporal delay, increased up to 4.5 h as the ionic strength and halide polarizability increased. Another study by the Bonn group highlighted the effect of flow on the silica–water interface, which seemed to reversibly alter the balance between the dissolution of silica and deprotonation of surface silanol groups.^[5]^ Under neutral conditions and 10 mm background electrolytes, they observed a drop in the ssp SFG intensity of water upon flow, which recovered on a timescale of 30 minutes. They assigned this drop to a lowering of the effective surface charge as a result of a fast hydrolysis of silica compared to a slower deprotonation of the silanol groups under these conditions. In a follow‐up ssp SFG investigation, Bonn and co‐workers determined that the interfacial concentration of dissolved silica saturates in the millimolar range over a timescale of tens of hours. Moreover, the observed kinetics indicated that dissolution is an autocatalytic process.^[69b]^ The notion of a shift in the dissolution equilibrium as a result of flow seems a generic property of mineral–water interfaces: as already mentioned in the CaF_2_ section, this mineral flow can also affect the interfacial equilibrium.^[5]^ As fluoride dissolves more readily than calcium at an acidic pH value, the surface will become charged and fluoride ions will be present in the near‐surface region. Upon flow, the fluoride concentration in the near‐surface region is modified, which influences the dissolution equilibrium.

#### 8.2.2 Freezing at the Mineral–Water Interface

Ice formation in the atmosphere occurs through heterogeneous nucleation, as homogeneous nucleation of ice cannot occur until temperatures below −40 °C are reached. Mineral dust particles play a major role in the heterogeneous nucleation of ice,^[71]^ with different minerals displaying very different ice‐nucleating capabilities. For example, ice formation on the surface of feldspar particles was found to be remarkably efficient.^[71]^ A combination of in situ scanning electron microscopy and molecular dynamic simulations revealed that nucleation occurs on specific defect sites of the feldspar surface.^[72]^ The question is, what are the underlying “rules” for efficient heterogeneous nucleation by minerals? To answer this, several groups[^65d^, ^73^] in the past years have studied changes in the SHG response and SFG spectrum upon the freezing of water at mineral–water interfaces to understand the molecular‐level details of this phase transition relevant for, for example, atmospheric processes. In general, and in agreement with an SHG study,^[65a]^ no change in the water structure is observed upon cooling as long as the water is liquid. Different groups have reported different effects when the water freezes. In 2015, Leisner and co‐workers performed temperature‐dependent SHG measurements of the water–muscovite (001) interface.^[65a]^ They observed, in contrast to the sapphire–water interface, a substantial change in the SHG response far above the freezing point of water, which they interpreted as the preordering of interfacial water and facilitation of ice nucleation by the mica surface compared to the poor ice nucleator sapphire. A follow‐up study discussed freezing mechanisms under various conditions.^[65b]^

By comparing the freezing temperature with the amplitude of the ssp SFG signal in the liquid state, Bonn and co‐workers^[74]^ found that increasing the surface charge of the alumina (0001) surface through variation of the bulk pH value shifts freezing to a lower temperature and, therefore, suppresses ice nucleation. In turn, heterogeneous nucleation seemed to be most efficient at a neutral alumina surface, that is, around a neutral pH value, where the surface does not dictate the ordering of the interfacial water molecules. Moreover, a joint SFG, simulation, and nucleation‐temperature study on the mica–water interface grafted with different positive ions has shown that, also here, the ice nucleation ability depends on the water ordering at the interface.^[65e]^ A reduced ordering of interfacial water was found to correlate with higher ice nucleation temperatures. This conclusion is in line with a temperature‐dependent SFG study, using both ssp and ppp, from Dhinojwala and co‐workers^[65d]^ on the mica–water interface. They concluded that the orientation of water molecules next to the surface plays an important role in the structure of ice. SFG experiments (ssp) on the mica–water interface with concentrations of sulfuric acid between 0.5 and 5 m showed a decreasing SFG signal with an increasing concentration of acid, which was interpreted as a reduced water ordering.^[65c]^ The authors linked their results to the observation of higher acid concentrations resulting in poorer ice nucleation activity. They concluded that, apparently, structured water is needed for the efficient heterogeneous nucleation of ice. This seems to contradict the discussion above that reduced water ordering was associated with enhanced ice nucleation. However, at these high sulfate concentrations, sulfate may be absorbed at the surface and/or almost all the water molecules might be involved in the hydration of the sulfate ions, thereby resulting in a lack of free water.

### 8.3 Summary

In this Review, we have summarized the extensive efforts to study mineral–water interfaces with second‐order nonlinear spectroscopy that started about 30 years ago and remains of increasing interest. By exploiting the large number of experimental degrees of freedom that SFG and SHG offer, this field has provided a variety of unique insights into these interfaces. For the three oxide interfaces silica, alumina, and titanium dioxide, the surface charge can be regulated by pH‐dependent deprotonation and/or protonation. As the pzc for silica is around pH 2, the surface is negatively charged at a near‐neutral pH value; for the other two surfaces, both positively and negatively charged surfaces are possible at typical pH values, as the pzc is around 6. As the surface charge is dependent on the pH value, the second‐order nonlinear signals also vary with pH. Especially for silica, the research of the last years has focused on understanding what these spectroscopic techniques measure. Consensus has been reached that for the charged silica surface, a major part of the signal in the hydrogen‐bonded OH stretch region originates from bulk *χ* ^(3)^ contributions. Increasing electrolyte concentrations reduces the signal strength mainly through screening, but ion‐specific effects are also observed. For charged titanium dioxide at low and high pH values, the contribution of the bulk *χ* ^(3)^ signal does not seem to dominate the response. For certain alumina interfaces, a response in the hydrogen‐bonded region is also observed, as well as a high‐frequency mode close to 3700 cm^−1^, which is assigned to hydroxy groups. For silica, in contrast, such a high‐frequency mode has only been observed after special heat treatment of the surface. The CaF_2_ surface is charged under low pH conditions due to the dissolution of fluoride. As a result, the water molecules orient to the interface and result in a large second‐order optical response. At high pH values, fluoride exchanges with hydroxy groups and results in CaOH groups. Although the discussed minerals all have different chemical structures, many of the properties of the interfacial water structure seem dominated by the charge present on the mineral and much less by its chemical composition. For interfacial chemistry, the detailed interfacial composition of the mineral is, of course, crucial.

We hope to have shown here that research in the field of nonlinear optical studies of mineral–water surfaces has progressed to the point where we can start to address geochemically and atmospherically relevant questions pertaining to interfacial chemistry under non‐equilibrium conditions and, for example, the mechanisms underlying the heterogeneous nucleation of ice.

## Conflict of interest

The authors declare no conflict of interest.

## Biographical Information

*Ellen Backus is professor for physical chemistry at the University of Vienna and a group leader at the Max Planck Institute for polymer research in Mainz. Her research focuses on the molecular structure and (ultrafast) dynamics of various aqueous interfaces by using surface‐specific vibrational spectroscopy. She obtained her PhD in 2005 at the Leiden University with Prof. M. Bonn and A. W. Kleyn. After postdoctoral research at the University of Zurich and at AMOLF in Amsterdam with Prof. P. Hamm, she started in Mainz in 2012 and started in Vienna in 2018*.

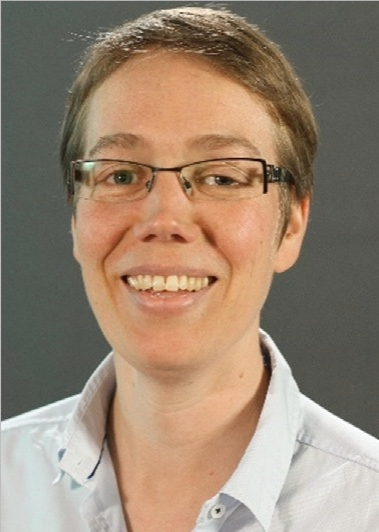

## Biographical Information

*Jan Schäfer received his PhD in 2019 from the University of Mainz for his work at the Max Planck Institute for Polymer Research. His studies focused on sum frequency generation spectroscopy experiments on non‐equilibrium aqueous interfacial systems. Before that, he received his Master degree at Ruhr University of Bochum for spectroscopic studies of water clusters with organic radicals in ultracold helium nanodroplets. Currently, he is affiliated with Coherent Shared Services B.V.*.

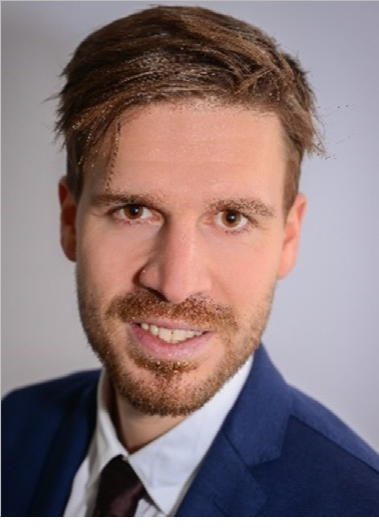

## Biographical Information

*Mischa Bonn is Max Planck Director at the Institute for Polymer Research (MPIP) in Mainz, Germany, where he heads the Department of Molecular Spectroscopy. He completed his PhD at the FOM‐Institute for Atomic and Molecular Physics (AMOLF) in Amsterdam (Profs. Kleyn/Van Santen), followed by postdoctoral positions at the Fritz‐Haber Institute in Berlin (Prof. Ertl) and Columbia University in New York (Prof. Heinz). After faculty appointments at AMOLF and Leiden University, the central theme of his research at MPIP is the characterization and control of the structure and dynamics of molecules, specifically at interfaces*.

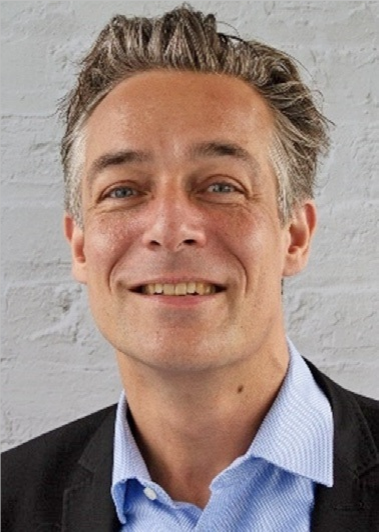

## Supporting information

As a service to our authors and readers, this journal provides supporting information supplied by the authors. Such materials are peer reviewed and may be re‐organized for online delivery, but are not copy‐edited or typeset. Technical support issues arising from supporting information (other than missing files) should be addressed to the authors.

Supplementary
