# Supplementary material for: Probing the Mineral–Water Interface with Nonlinear Optical Spectroscopy
Source: Angew Chem Int Ed Engl. 2020 Dec 9;60(19):10482–501. doi: 10.1002/anie.202003085 (PMC8247323; doi:10.1002/anie.202003085)
Supplement: Supplementary file 1 — Supplementary [file ANIE-60-10482-s001.pdf]

Angewandte Chemie
Eine Zeitschrift der Gesellschaft Deutscher Chemiker

Supporting Information

# Probing the Mineral–Water Interface with Nonlinear Optical Spectroscopy

*Ellen H. G. Backus*[+],* *Jan Schaefer*[+], *and Mischa Bonn**

anie_202003085_sm_miscellaneous_information.pdf

# Overview table

| Material | Surface type | Chemical environment | | Ref. | Method | Geometry | Result |
|---|---|---|---|---|---|---|---|
| **Silica** | Fused | Ion-dependence | @pH 5.8<br>@pH 10<br>0.01-0.1 M<br>NaCl, LiCl | Eisenthal, 1992, [1] | SHG (@300 nm) | EW | • Signal decreases with increasing salt concentration<br>• Independent of cation identity |
| | | | @pH 5.7<br>0-0.1 M<br>NaCl, KCl, LiCl | Chou, 2009, [2] | SFG (OH stretch), ssp | SA (window Backside) | • Concentration sensitivity of the signal on the order $K^+ > Li^+ > Na^+$<br>• Double band feature (3200 $cm^{-1}$ and 3400 $cm^{-1}$<br>• 3200 $cm^{-1}$ band is more concentration sensitive |
| | | | @pH 6.5<br>0-0.12 M<br>NaCl | Hore, 2009, [3] | SFG (OH stretch) ssp, sps | EW | • 2-3 features in the band<br>• Spectral weight on the low-frequency side for ssp, on the high-frequency side for sps<br>• With increasing concentration, the overall signal, the ratio between low- and high-frequency band and the sps/ssp ratio decrease |
| | | | @pH 6<br>$4.8*10^{-5}$-4.1 M<br>NaCl | Hore, 2011, [4] | SFG (OH stretch) ssp | EW | • 4 distinct regimes of monotonically decreasing signal with increasing ion concentration:<br>A: c < 0.5 mM: Plateau<br>B: 0.5 mM < c < 100 mM: Decrease<br>C: 0.1 M < c < 1 M: Plateau<br>D: c > 1 M: Decrease |

| | | | | | | | |
|---|---|---|---|---|---|---|---|
| | | | @pH 6<br>$10^{-4}$ – 0.5 M<br>NaCl | Borguet, 2011, [5] | SFG (OH stretch)<br>ssp<br>t-resolved | EW | • Vibrational lifetime increases with increasing ion concentration from ~200 fs at low to ~700 fs at high concentration |
| | | | @pH 10 and 6.5<br>0.1 mM:<br>CsCl, RbCl, LiCl, $NH_4Cl$, KCl, NaCl<br>0.033 M:<br>$CaCl_2$, $MgCl_2$, $ZnCl_2$ | Cremer, 2012, [6] | SFG (OH stretch) | SA | • Signal intensity shows a direct Hofmeister trend from $ZnCl_2$ giving the weakest response to LiCl with the largest signal |
| | | | @pH 6<br>$10^{-7}$ – 4 M<br>NaCl<br>LiCl | Backus 2017, [7] | SFG (OH stretch)<br>ssp | SA | • Under SA geometry, the signal decreases with decreasing concentration within regime A of the Hore work<br>• This results in a maximum signal around mM concentration<br>• The maximum position seems to be dependent on the cation size |
| | | | @pH 7<br>$10^{-5}$ – 0.5 M<br>NaCl<br>(CsCl, RbCl<br>KCl, NaBr, NaI) | Eisenthal 2018, [8] | SHG (@400 nm)<br>p-in + all-out,<br>s-in + all-out | EW | • Qualitatively the same curve shape<br>• Shape independent of polarization combination and salt species<br>• Curve maximum varies by 20% depending on the scanning direction (low-to-high vs. high-to-low conc.) |

| | | | | | | | |
|---|---|---|---|---|---|---|---|
| | | | @pH 12<br>1.1 -5 M<br>NaCl<br>Isotopic dilution | Tahara 2018, [9] | SFG (OH stretch)<br>ssp<br>phase-resolved | SA | • Signal decrease with increasing ion concentration (up to 1 M), until it saturates<br>• 2 broad main features<br>• At low ion concentration, the 3200 $cm^{-1}$ feature vanishes upon isotopic dilution |
| | | | @ pH 7, pH 5.8<br>$10^{-5}$ – 1 M<br>(NaCl, NaBr, NaI and NaCl, KCl, RbCl, CsCl) | Geiger, 2019, [10] | SHG (@400 nm)<br>s-in + all-out<br>+ phase resolved | EW | • Both, cation and anion size does not affect the curve shape and maximum position except for the plateau at high concentration which increases with decreasing ion size |
| | | | @pH 6<br>$10^{-7}$ – $5*10^{-2}$ M<br>NaCl<br>Angular dependence<br>(40.4°-74°) | Hore and Tyrode 2019, [11] | SFG (OH stretch)<br>ssp | SA, EW | • Well below the critical angle, the SFG signal drops below 0,1 mM salt concentration<br>• Close to the critical angle, a plateau is observed at this regime<br>• Results are rationalized with a model that considers 2nd and 3rd order contributions to the SFG response and an electrostatic potential beyond Debye Hückel approximation<br>• Proposed model yields a global fit for angular dependence of the SFG signal as a function of ion concentration |

| | | | | | | | |
|---|---|---|---|---|---|---|---|
| | | | T-dependence (10°C – 75°C) | Hore 2020, [12] | SFG (OH stretch) ssp | EW | • The intensity of the OH stretch band increases with increasing temperature from 10°C to 64°C and decreases from 64°C to 75°C<br>• This observation is rationalized as a convolution between increasing surface charge and decreasing contribution of aligned water molecules to the overall response |
| | | pH-dependence | @0.5 M (NaCl) pH 2 - 14 | Eisenthal 1992, [1] | SHG (@300 nm) | EW | • Monotonic signal increase with increasing pH<br>• 2 turning points at pH 4.5 and pH 8.5 |
| | | | No salt added pH 1.5 - 11 | Shen 2004, [13] | SFG (OH stretch) ssp | SA | • Low-frequency feature varies with pH similar to the SHG results, high-frequency feature almost insensitive to pH variation |
| | | | No salt added @pH 5.7 | Shen 2006, [14] | SFG (OH stretch) ssp t-resolved | EW | • Vibrational lifetime of OH stretch is ~300 fs (similar to bulk water) |
| | | | pH 2, pH 12 | Borguet 2009, [15] | SFG (OH stretch) ssp t-resolved | EW | • Vibrational lifetime slow at pH 2 (~570 fs) and bulk-like at pH 12 (~255 fs) |
| | | | | | | | • |

| | | | | | | | |
|---|---|---|---|---|---|---|---|
| | | | pH 2, pH 12<br>Isotopic dilution | Borguet 2010, [16] | SFG (OH stretch)<br>ssp<br>t-resolved | EW | • The vibrational lifetime is longer at the high-frequency feature of the band at both pH conditions<br>• From neutral conditions towards both pH directions, the spectral weight shifts red |
| | | | @0.5 M<br>Alkali Chlorides<br>pH 2 - 12 | Gibbs 2012, [17] | SHG (@275 nm)<br>s-in+ all-out | EW | • Bimodal titration curve<br>• The high pH inflection point shifts due to the presence of different salts:<br>pH 8.3 (NaCl) to pH 10.8 (LiCl) |
| | | | @0.1 M<br>NaCl<br>pH 2 - 12 | Borguet 2013, [18] | SFG (OH stretch)<br>ssp | EW | • The SFG response is most sensitive to addition 0f 0.1 M NaCl around neutral pH and least sensitive at low and high pH |

| | | | | | | | |
|---|---|---|---|---|---|---|---|
| | | | @0.5 M<br>$Na^+$, $K^+$ halides<br>pH 2 – 12<br><br>@0.5 M NaI<br>pH 2 – 12 | Gibbs 2013, [19] | SHG (@275 nm)<br>s-in+ all-out<br><br>SFG (OH stretch)<br>ppp | EW<br><br>EW | • With increasing halide size, the titration curve gets sharper<br><br>• In contrast to SHG results, the SFG signal increases towards acidic and alkaline pH<br>• At neutral pH, the Signal decrease by a factor of 30-40 when adding 0.5 M salt to pure water<br>• This is much higher than reported by the Hore group for the ssp response and opposite to the SHG results which show a 2.5 fold increase upon adding 0.5 M salt |
| | | | @0.1 vs 0.5 M Alkali chlorides and iodides<br>pH 2 - 12 | Gibbs 2014, [20] | SHG (@275 nm)<br>s-in+ all-out | EW | • Titration curve gets more similar when the concentration is reduced to 0.1 M<br>• Curve shifts induced by different halide species are merely affected by concentration effects |
| | | | @10 mM NaCl<br>pH 2-12 | Gibbs 2015, [21] | SHG (@275 nm)<br>p-in+ all-out | EW | • Titration curve shifts and shows 2 or 3 steps depending on the starting pH:<br>From pH 2 or 7 $\rightarrow$ 2 steps<br>From pH 12 $\rightarrow$ 3 steps |
| | | | @10 mM NaCl<br>pH 3, 6, 10<br>Surface treatment: Plasma vs heat | Tyrode 2017, [22] | SFG (OH stretch)<br>ssp, ppp, sps | EW | • Presence of a third (high-frequency feature) at ~ 3650 cm$^{-1}$, which indicates a weakly interacting OH group<br>• This feature is enhanced for heat-treated silica |

| | | | | | | | |
|---|---|---|---|---|---|---|---|
| | | | @10 mM NaCl<br>pH 6<br>Non-heated vs heated surface | Backus 2019, [23] | SFG (OH stretch)<br>ssp, ppp<br>static spectra vs.<br>phase-resolved<br>t-resolved | <br><br>SA<br>SA<br>EW | • The weakly interacting OH groups are oriented towards the silica surface |
| | | | @2, 4, 6, 12 M of chlorides of $Na^{+}$, $Li^{+}$ and $Mg^{2+}$, $Ca^{2+}$<br>~neutral pH | Chou 2016, [24] | SFG (OH stretch)<br>ssp | EW | • At high concentration, the double band feature breaks into one low intensity, high-frequency band around 3500 $cm^{-1}$ |
| | | | @10mM phosphate buffer<br>pH 2.1, 5.6, 7.2 and 12.1<br>isotopic dilution | Tahara 2016, [25] | SFG (OH stretch)<br>ssp<br>phase-resolved | SA | • At neutral pH, 3200 $cm^{-1}$ feature disappears indicating vibrational coupling<br>• Uncoupled spectrum still varies in shape and intensity with pH, owing to positive and negative contributions at high and low frequency, respectively<br>• The red part is negative at low pH and flips towards high pH |
| | | | @0.01, 0.05 and 0.1 M NaCl<br>pH 2 - 12 | Gibbs 2017, [26] | SFG (OH stretch)<br>ssp vs pss | EW | • Titration curve under pss is similar to what is known for SHG<br>• For ssp, the titration curve is non-monotonic with a minimum around neutral pH and increase towards low and high pH<br>• This trend is more pronounced at higher NaCl concentration (0.1 M) |
| | | | @0.5 M alkali chlorides<br>pH 2 - 12 | Gibbs 2017, [27] | SFG (OH stretch)<br>ssp | EW | • Signal intensity at neutral pH increases in the series:<br>$Cs^{+} < K^{+} < Na^{+} < Li^{+}$<br>• This trend is reversed at low/high pH |

| | | | | | | | |
|---|---|---|---|---|---|---|---|
| | | | @0.5 M NaCl | Hore and Gibbs 2019, [28] | SFG (OH stretch)<br>ssp, ppp, pss<br>SHG (@400 nm) | EW | • SHG increases monotonically with increasing pH while SFG shows a minimum at around neutral pH<br>• Conclusion: SHG is dominated by the surface potential and SFG is more sensitive to the SFG activity of sub-ensembles of differently oriented water species |
| | | | @0, 0.1, 0.5 M NaCl<br>pH 2, 6, 12 | Borguet 2020, [29] | SFG (OH stretch)<br>ppp<br>t-resolved | EW | • Acceleration of interfacial vibrational energy relaxation due to kosmotropic effect of ions driving the in-plane ordering of water at the topmost interfacial layer<br>• Formation of local hydrophobic areas on the silica interface |

| | | | | | | | |
|---|---|---|---|---|---|---|---|
| | | Chemistry | @0.01 – 0.5 M alkali halides pH titration | Geiger 2008, [30] | SHG (@300 nm) p-in + all-out | EW | • SHG response (as reporter of the surface conditions) lags bulk pH changes both, spatially and temporally<br>• Delay time increases with ion concentration (up to ~4.5 h) and with polarizability of the halide |
| | | | @0.01 M NaCl Effect of flow | Bonn 2014, [31] | SFG (OH stretch) ssp | EW | • At neutral pH, flow drops the SFG signal<br>• Recovery time with flow-off about 0.5 h<br>• At low and high pH, flow does not have an effect on the SFG response |
| | | | @$10^{-7}$ to 3 M NaCl, pH~6.5 Equilibration | Bonn 2018, [32] | SFG (OH stretch) ssp | SA | • Within roughly a day, the SFG response of pure water rises to an equilibrated level<br>• This level corresponds to addition of $5*10^{-4}$ – $10^{-2}$ M of salt (NaCl)<br>• Upon flow, the signal drops again |

| | | | | | | | |
|---|---|---|---|---|---|---|---|
| | α-Quartz | pH-dependence | pH 1.5 – 12.3<br>0.1, 0.5 M NaCl | Shen 1994, [33] | SFG (OH stretch)<br>ssp, ppp, pss, sps | SA | • Double feature (3200, 3450 $cm^{-1}$) with different relative and absolute intensity depending on pH, ion concentration and polarization combination<br>• From low to high pH (without salt), the absolute intensity decreases first and increases when passing pH 5.6 |
| | | | No salt added<br>pH 1.5 - 11 | Shen 2004, [13] | SFG (OH stretch)<br>ssp | SA | • With increasing pH, the signal increases monotonically<br>• Compared to fused silica, the high-frequency band is more pronounced |
| | | | No salt added<br>pH 1.5 - 11 | Shen 2005, [34] | SFG (OH stretch)<br>ssp<br>phase-resolved | SA | • The absolute spectrum is composed of two positive high frequency and one negative low-frequency feature<br>• The negative feature decreases with increasing pH<br>• The positive features increase with increasing pH |

| | | | | | | | |
|---|---|---|---|---|---|---|---|
| | Sapphire | | No salt added<br>pH 3 – 12<br>dehydrated vs. hydrated surface | Pink 1999, [35] | SFG (OH stretch)<br>ppp | EW | • 3 featured OH stretch band<br>• Overall response enhanced by a factor of ~6 if the surface is hydrated<br>• At the isoelectric point (pH 8) the signal is lowest and increases towards lower and higher pH with changing relative sign |
| | | | T-dependent | Leisner 2015, [36] | SHG (@400 nm) | EW | • Small decrease in SHG signal upon cooling down till the phase transition<br>• At phase transition stepwise large decrease in SHG intensity upon freezing |
| **Alumina** | Corundum | | @ 1-100 mM of NaCl, $NaNO_3$, $NaClO_4$<br>pH 2 - 12 | Eggelston 2001, [37] | SHG (@520 nm) | EW | • Point of zero charge is between pH 5 and 6 |
| | Corundum (0001) vs. $(1\bar{1}02)$ | | @ 1, 10 and 100 mM of $NaNO_3$<br>pH 4 – 7.5<br><br>@pH 6.7, 4<br>1 – 100 mM | Eisenthal 2005, [38] | SHG (@400 nm)<br>p-in + p-out | SA | • Titration curves indicate pzc at pH 4.1 ± 0.4 for (0001) and pH 5.2 ± 0.4 for (1102)<br><br>• With increasing ion concentration, the signal decreases above the pzc and increases below the pzc |
| | Corundum (001) | | pH 2.7 – 12 | Fanghänel 2008, [39] | SFG (OH stretch)<br>ssp, ppp | EW | • Above 3400 $cm^{-1}$ 4 doubly coordinated ($Al_2OH$) surface OH species<br>• Below 3400 $cm^{-1}$ at pH 12 two broad resonances due to water molecules oriented with the H to the surface |

| | | | | | | | |
|---|---|---|---|---|---|---|---|
| | Corundum ($1\bar{1}02$) | | pH 2.7 – 10.5 | Waychunas 2011, [40] | SFG (OH stretch)<br>ssp, sps<br>+ phase resolved | SA | • Point of zero charge at pH ~ 6.7<br>• Bands at ~3230 and ~3490 $cm^{-1}$ associated with interfacial water molecules and H-bonded hydroxyls associated with $AlOH_2$ groups<br>• pK values of 4.9 an 9.2 for protonation/deprotonation of $Al_3OH$ and $AlOH_2$ groups |
| | Amorphous and (0001) | | pH 3.5 -10.1<br>no salt vs<br>50 mM NaCl | Shen 2008, [41] | SFG (OH stretch)<br>ssp, ppp, sps<br>+ phase resolved | SA | • Spectrum shows typical double feature plus a predominant band in the “free-OH” region<br>• From polarization combination, average tilt angle of ~26° are deduced for the free-OH<br>• pzc is found around pH 6.3 which is lower than for amorphous alumina or powder<br>• Addition of salt reduces H-bonded signal significantly, if pH far away from the pzc |
| | (0001) | | Surface roughness<br>pH 0.8 – 14 | Daum 2008, [42] | SFG (OH stretch)<br>ssp | SA | • Surface disorder affects molecular structure<br>• On a nanorough surface presence of uncoupled hydroxyls groups, presumably in hydrophobic nanopores<br>• Different pK value for deprotonation of surface hydroxyl groups at smooth and rough surface |

| | | | | | | | |
|---|---|---|---|---|---|---|---|
| | | | Surface preparation<br>Water vapor | Campen 2015, [43] | SFG (OH and Al-O stretch)<br>ssp | | • Hydroxylated surface in ambient air shows a 3700 $cm^{-1}$ band, while this band is absent for the 1-Al terminated surface prepared in UHV |
| | $(11\bar{2}0)$ | | pH 2.5 – 11<br>no salt vs 50 mM | Waychunas, 2012 [44] | SFG (OH stretch)<br>phase-resolved | SA | • Sign change of $\mathrm{Im}\chi^{(2)}$ spectrum indicates pzc for pH ~ 6.7<br>• Upon adding salt signal decrease at low and high pH<br>• Spectral changes due to protonation and deprotonation |
| | | | pH 2 – 12<br>no salt vs 0.1 and 0.5 M NaCl | Borguet 2016, [45] | SFG (OH stretch)<br>ssp and ppp<br>static spectra<br>t-resolved | EW | • ~3200 and ~3400 $cm^{-1}$ attenuated by the addition of salt<br>• Largely salt concentration-independent feature at 3000 $cm^{-1}$ assigned to surface aluminol groups and/or interfacial water molecules strongly H-bond to alumina<br>• Fast (bulk waterlike) vibrational relaxation dynamics, insensitive to changes in surface charge and ionic strength |
| | (1120) | | pH 13, KOH | Borguet 2014, [46] | SFG (OH stretch)<br>Free Induction Decay | EW | • OH oscillators from interfacial water, centered at 3679 $cm^{-1}$, dephase on a ~90 fs timescale<br>• Dephasing of interfacial alumina hydroxyls with a resonance at 3644 $cm^{-1}$, is an order of magnitude slower |

| | | | | | | | |
|---|---|---|---|---|---|---|---|
| | 0001 vs. $(11\bar{2}0)$ | | pH 2 – 12<br>no salt vs 0.1 M NaCl | Borguet 2017, [47] | SFG (OH stretch)<br>ppp<br>static spectra<br>t-resolved | EW | • H-bonded OH stretch is blue-shifted for 0001 compared to $(11\bar{2}0)$<br>• For neutral alumina, the dynamics are slower by a factor of 2 for 0001 compared to $(11\bar{2}0)$<br>• For charged 0001, the dynamics are faster than in bulk or at charged silica<br>• The addition of salt has little to no effect on the dynamics |
| | Sapphire (0001) | | pH 3 – 9<br>T-dependent | Bonn 2017, [48] | SFG (OH stretch)<br>ssp | EW | • Upon freezing, the 3200 $cm^{-1}$ band increases and the 3400 $cm^{-1}$ band decreases<br>• At neutral surface (~pH 7) ice nucleation is most efficient |
| | | | pH 4, 10<br>halide ions | Borguet 2018, [49] | SFG (OH stretch)<br>ppp<br>static spectra<br>t-resolved | EW | • Attenuation of SFG signal at positively charged surface followed the sequence $F^->>Br^->Cl^->I^-$<br>• At negative surface attenuation follows $Br^->I^-\approx Cl^->F^-$<br>• Only $F^-$ perturbs the vibrational lifetime of water next to positively charged surface |
| | | | Monovalent vs divalent cations<br>pH 4, 6, 10 | Borguet 2019, [50] | SFG (OH stretch)<br>ppp<br>static spectra<br>t-resolved | EW | • Lower binding affinity at the interface for monovalent compared to divalent cations<br>• At pH 10, negatively charged surface, addition of divalent cations increases spectral intensity at 3400 $cm^{-1}$; monovalent cations only attenuated SFG intensity |

| | | | | | | | |
|---|---|---|---|---|---|---|---|
| | | | | | | | • Presence of NaCl and $BaCl_2$ does not change the vibrational lifetime |
| **Calcium Fluoride** | | | pH dependent, with addition of NaF | Richmond 2001,[51] | SFG (OH stretch) ssp | EW | • The signal is largest under acidic conditions and vanishes around neutral pH<br>• At basic pH the signal increases again and an additional free-OH feature appears<br>• Upon addition of NaF, this free-OH band decreases |
| | | | @pH 5.8<br>$10^{-5}$ – 1 M of $Na^+$ and $Ca^{2+}$ salts | Richmond 2010, [52] | SFG (OH stretch) | EW | • Signal is more sensitive to the presence of NaF and $Na_2SO_4$ than to that of NaCl and NaBr |
| | | | @pD 3.7 | Benderskii 2005, [53] | SFG (OD stretch) ssp<br>Free Induction Decay | SA | • Exciting the weaker hydrogen bond ensemble shows monotonic decay and shift to lower frequency on a 100 fs time scale<br>• Exciting stronger hydrogen bond ensemble results in shift to higher frequency and time scale of 125 fs<br>• Dynamical heterogeneity of water hydrogen bonding network |
| | | | no salt added<br>@pH 3, 9.5, 12<br>Water flow vs resting | Bonn 2014, [31] | SFG (OH stretch) ssp | EW | • signal is large under acidic conditions, minimum around neutral pH and increases again towards alkaline conditions<br>• Upon flow, signal increases at low pH, decreases at high pH and is only affect in spectral shape around neutral pH |

| | | | | | | | |
|---|---|---|---|---|---|---|---|
| | | | no salt added<br>pH 2, 7, 13 | Sulpizi 2016, [54] | SFG (OH stretch)<br>phase-resolved | SA | • Large signal at pH 2<br>• Lower signal at higher pH values<br>• pH 13: A "free OH" band is present |
| | | | No salt added | Borguet 2016, [55] | SFG (OH stretch)<br>ppp<br>Free Induction Decay | EW | • two featured bands at 3140 $cm^{-1}$ and 3410 $cm^{-1}$ show a 70 fs and 50 fs dephasing time, respectively |
| | | | Flow perturbed @different flow rates<br>(with varying pH and salt concentration | Abdelmonem, 2018<br>[56] | SHG (@400 nm)<br>ssp, ppp | EW | • The SHG response for the flow perturbed steady-state increases with increasing flow rate |
| | | | pD/pH 2 | Sulpizi and Backus 2019, ([57]) | Time-dependent 2D-SFG (OD/OH stretch)<br>ssp | EW | • Fast vibrational relaxation and spectral diffusion for strongly hydrogen-bonded OD groups |
| **Titanium Dioxide** | Thin film on fused silica: 0.9 – 3.9 nm | | @30mM salt<br>(NaCl vs. PBS)<br>pH 2 - 12 | Cremer 2004, [58] | SFG (OH stretch)<br>ssp | SA | • Typical double featured band<br>• With NaCl: SFG minimum at pH 4-6 (known as the isoelectric point of $TiO_2$)<br>• Towards lower/higher pH, the 3200 $cm^{-1}$ band increases relative to the 3400 $cm^{-1}$ band<br>• PBS buffer shift the minimum to pH 2, similar to titration curve for silica |

| | | | | | | | |
|---|---|---|---|---|---|---|---|
| | Thin film on fused silica | | Water vapor on UV-irradiated surface | Nihonyanagi 2004, [59] | SFG (OH stretch) ppp | EW | • UV-irradiation increases the H-bonded OH-stretch signal substantially as well as a third “free-OH” feature |
| | Thin film on quartz:<br>1 nm | | @ pH 10,<br>With 0.33 and 0.033 mM of: CsCl, RbCl, LiCl, $NH_4Cl$, KCl, NaCl $CaCl_2$, $MgCl_2$, $ZnCl_2$ | Cremer 2012,[6] | SFG (OH stretch) | SA | • Signal intensity is in direct relation to the Hofmeister trend from $ZnCl_2$ giving the weakest response to CsCl with the largest signal |
| | Thin film on $CaF_2$:<br>50 – 200 nm | | Isotopic dilution | Backus 2017, [60] | SFG (OH stretch) ssp<br>static spectra<br>phase-resolved | SA | • Double feature: 2 bands of opposite sign<br>• Negative high-frequency feature and positive low-frequency feature<br>• Shape changes upon isotopic dilution |
| | Thin-film on $CaF_2$:<br>85, 150 nm | | @0, 10, 100 mM of NaCl<br>pD 3 - 11 | Backus 2019, [61] | SFG (OD stretch)<br>Static spectra<br>Phase-resolved | SA (Two VIS angles) | • Intensity minimum at pD 5<br>• Spectral differences from sample to sample<br>• High-frequency part of the double band flips sign when pD crosses pzc, low-frequency part keeps sign across full pD range |

| | | | | | | | |
|---|---|---|---|---|---|---|---|
| **Mica** | Cleaved | | Water vapor on the surface | Salmeron 1998, [62] | SFG (OD stretch) ssp | SA | • With increasing humidity, the typical double feature arises with the low-frequency band being predominant<br>• Also, a “free OD” is present |
| | Cleaved | | pH-dependence using $D_2SO_4$ | Chou 2011,[63] | SFG (OD stretch) ssp | SA | • SFG signal decreasing with the addition of $D_2SO_4$ and is drastically diminished from c > 0.5 M |
| | Muscovite (001) With IMG fixed on sapphire | | T-dependent | Leisner 2015, [36] | SHG (@400 nm) | EW | • The non-linear properties change way before freezing point |
| | Adhered onto a sapphire prism | | T-dependent and ice formation | Dhinojwala, 2016 [64] | SFG (OH stretch) ssp, ppp | EW | • Upon freezing, the water signal in front of a mica surface drops<br>• The hysteresis of this drop is about 10°C between cooling and heating the cycles |
| | Cleaved (001) | | T-dependent and ice formation | Abdelmonem, 2017 [65] | SHG (@400 nm) SM polarization | EW | • The SHG signal drops upon formation of a thin film of water o the mica surface, regardless of the formed phase und freezing path |
| | Muscovite (001) | | Azimuthal angle dependence | Wang 2019, [66] | SFG (OH stretch) | SA: multiple layer reflectio ns | • Signal highly anisotropic<br>• SFG can be used to discriminate bottom from top mica face |
| | Muscovite (001) | | Cationic termination: Cleaved, $H^+$, $Na^+$, $K^+$, $Cs^+$ | Zeng, Bonn and Wang, [67] | SFG (OH stretch) | SA | • H-mica induces less orientational order on interfacial water than Na- and K-mica as indicated by a lower SFG response in the OH stretching band |

## References


[1] S. Ong, X. Zhao, K. B. Eisenthal, *Chem. Phys. Lett.* **1992**, *191*, 327-335.

[2] Z. Yang, Q. Li, K. C. Chou, *The Journal of Physical Chemistry C* **2009**, *113*, 8201-8205.

[3] K. C. Jena, D. K. Hore, *J. Phys. Chem. C* **2009**, *113*, 15364-15372.

[4] K. C. Jena, P. A. Covert, D. K. Hore, *J. Phys. Chem. Lett.* **2011**, *2*, 1056-1061.

[5] A. Eftekhari-Bafrooei, E. Borguet, *J. Phys. Chem. Lett.* **2011**, *2*, 1353-1358.

[6] S. C. Flores, J. Kherb, N. Konelick, X. Chen, P. S. Cremer, *The Journal of Physical Chemistry C* **2012**, *116*, 5730-5734.

[7] J. Schaefer, G. Gonella, M. Bonn, E. H. G. Backus, *Phys. Chem. Chem. Phys.* **2017**, *19*, 16875-16880.

[8] M. D. Boamah, P. E. Ohno, F. M. Geiger, K. B. Eisenthal, *J. Chem. Phys* **2018**, *148*, 222808.

[9] S.-h. Urashima, A. Myalitsin, S. Nihonyanagi, T. Tahara, *The Journal of Physical Chemistry Letters* **2018**, *9*, 4109-4114.

[10] M. D. Boamah, P. E. Ohno, E. Lozier, J. van Ardenne, F. M. Geiger, *The Journal of Physical Chemistry B* **2019**, *123*, 5848-5856.

[11] D. K. Hore, E. Tyrode, *J. Phys. Chem. C* **2019**, *123*, 16911-16920.

[12] M. S. Azam, C. Y. Cai, J. M. Gibbs, E. Tyrode, D. K. Hore, *J. Am. Chem. Soc.* **2020**, *142*, 669-673.

[13] V. Ostroverkhov, G. A. Waychunas, Y. R. Shen, *Chemical Physics Letters* **2004**, *386*, 144-148.

[14] J. A. McGuire, Y. R. Shen, *Science* **2006**, *313*, 1945-1948.

[15] A. Eftekhari-Bafrooei, E. Borguet, *J. Am. Chem. Soc.* **2009**, *131*, 12034-12035.

[16] A. Eftekhari-Bafrooei, E. Borguet, *J. Am. Chem. Soc.* **2010**, *132*, 3756-3761.

[17] M. S. Azam, C. N. Weeraman, J. M. Gibbs-Davis, *J. Phys. Chem. Lett.* **2012**, *3*, 1269-1274.

[18] S. Dewan, M. S. Yeganeh, E. Borguet, *J. Phys. Chem. Lett.* **2013**, *4*, 1977-1982.

[19] M. S. Azam, C. N. Weeraman, J. M. Gibbs-Davis, *J. Phys. Chem. C* **2013**, *117*, 8840-8850.

[20] M. S. Azam, A. Darlington, J. M. Gibbs-Davis, *J. Phys.-Condens. Mat.* **2014**, *26*, 244107.

[21] A. M. Darlington, J. M. Gibbs-Davis, *J. Phys. Chem. C* **2015**, *119*, 16560-16567.

[22] L. Dalstein, E. Potapova, E. Tyrode, *Phys. Chem. Chem. Phys.* **2017**, *19*, 10343-10349.

[23] J. D. Cyran, M. A. Donovan, D. Vollmer, F. Siro Brigiano, S. Pezzotti, D. R. Galimberti, M.-P. Gaigeot, M. Bonn, E. H. G. Backus, *Proceedings of the National Academy of Sciences* **2019**, *116*, 1520-1525.

[24] K. A. Lovering, A. K. Bertram, K. C. Chou, *The Journal of Physical Chemistry C* **2016**, *120*, 18099-18104.

[25] A. Myalitsin, S. H. Urashima, S. Nihonyanagi, S. Yamaguchi, T. Tahara, *J. Phys. Chem. C* **2016**, *120*, 9357-9363.

[26] A. M. Darlington, T. A. Jarisz, E. L. Dewalt-Kerian, S. Roy, S. Kim, M. S. Azam, D. K. Hore, J. M. Gibbs, *J. Phys. Chem. C* **2017**, *121*, 20229-20241.

[27] E. L. DeWalt-Kerian, S. Kim, M. S. Azam, H. Zeng, Q. Liu, J. M. Gibbs, *The Journal of Physical Chemistry Letters* **2017**, *8*, 2855-2861.

[28] B. Reh, M. Rashwan, E. L. DeWalt-Kerian, T. A. Jarisz, A. M. Darlington, D. K. Hore, J. M. Gibbs, *J. Phys. Chem. C* **2019**, *123*, 10991-11000.

[29] A. Tuladhar, S. Dewan, S. Pezzotti, F. Siro Brigiano, F. Creazzo, M. P. Gaigeot, E. Borguet, *J. Am. Chem. Soc.* **2020**, *142*, 6991-7000.

[30] J. M. Gibbs-Davis, J. J. Kruk, C. T. Konek, K. A. Scheidt, F. M. Geiger, *J. Am. Chem. Soc.* **2008**, *130*, 15444-15447.

[31] D. Lis, E. H. G. Backus, J. Hunger, S. H. Parekh, M. Bonn, *Science* **2014**, *344*, 1138-1142.

[32] J. Schaefer, E. H. G. Backus, M. Bonn, *Nat. Commun.* **2018**, *9*, 3316.

[33] Q. Du, E. Freysz, Y. R. Shen, *Phys. Rev. Lett.* **1994**, *72*, 238.

[34] V. Ostroverkhov, G. A. Waychunas, Y. R. Shen, *Phys. Rev. Lett.* **2005**, *94*, 46102.

[35] M. S. Yeganeh, S. M. Dougal, H. S. Pink, *Phys. Rev. Lett.* **1999**, *83*, 1179-1182.

[36] A. Abdelmonem, J. Lützenkirchen, T. Leisner, *Atmospheric Measurement Techniques* **2015**, *8*, 3519-3526.

[37] A. G. Stack, S. R. Higgins, C. M. Eggleston, *Geochimica et Cosmochimica Acta* **2001**, *65*, 3055-3063.

[38] J. P. Fitts, X. Shang, G. W. Flynn, T. F. Heinz, K. B. Eisenthal, *J. Phys. Chem. B* **2005**, *109*, 7981-7986.

[39] M. Florsheimer, K. Kruse, R. Polly, A. Abdelmonem, B. Schimmelpfennig, R. Klenze, T. Fanghanel, *Langmuir* **2008**, *24*, 13434-13439.

[40] J. H. Sung, L. N. Zhang, C. S. Tian, Y. R. Shen, G. A. Waychunas, *J. Phys. Chem. C* **2011**, *115*, 13887-13893.

[41] L. Zhang, C. Tian, G. A. Waychunas, Y. R. Shen, *J. Am. Chem. Soc.* **2008**, *130*, 7686-7694.

[42] B. Braunschweig, S. Eissner, W. Daum, *J. Phys. Chem. C* **2008**, *112*, 1751-1754.

[43] Y. J. Tong, J. Wirth, H. Kirsch, M. Wolf, P. Saalfrank, R. K. Campen, *J. Chem. Phys.* **2015**, *142*, 12.

[44] J. Sung, Y. R. Shen, G. A. Waychunas, *Journal of Physics-Condensed Matter* **2012**, *24*.

[45] A. Tuladhar, S. Dewan, J. D. Kubicki, E. Borguet, *J. Phys. Chem. C* **2016**, *120*, 16153-16161.

[46] A. Boulesbaa, E. Borguet, *J. Phys. Chem. Lett.* **2014**, *5*, 528-533.

[47] A. Tuladhar, S. M. Piontek, E. Borguet, *J. Phys. Chem. C* **2017**, *121*, 5168-5177.

[48] A. Abdelmonem, E. H. G. Backus, N. Hoffmann, M. A. Sánchez, J. D. Cyran, A. Kiselev, M. Bonn, *Atmos. Chem. Phys.* **2017**, *17*, 7827-7837.

[49] A. Tuladhar, S. M. Piontek, L. Frazer, E. Borguet, *J. Phys. Chem. C* **2018**, *122*, 12819-12830.

[50] S. M. Piontek, A. Tuladhar, T. Marshall, E. Borguet, *J. Phys. Chem. C* **2019**, *123*, 18315-18324.

[51] K. A. Becraft, G. L. Richmond, *Langmuir* **2001**, *17*, 7721-7724.

[52] A. J. Hopkins, S. Schrödle, G. L. Richmond, *Langmuir* **2010**, *26*, 10784-10790.

[53] A. N. Bordenyuk, A. V. Benderskii, *J. Chem. Phys.* **2005**, *122*, 134713.

[54] R. Khatib, E. H. G. Backus, M. Bonn, M. J. Perez-Haro, M. P. Gaigeot, M. Sulpizi, *Scientific Reports* **2016**, *6*, 1-10.

[55] A. Boulesbaa, E. Borguet, *J. Phys. Chem. Lett.* **2016**, *7*, 5080-5085.

[56] J. Lutzenkirchen, T. Scharnweber, T. Ho, A. Striolo, M. Sulpizi, A. Abdelmonem, *J. Colloid Interface Sci.* **2018**, *529*, 294-305.

[57] D. Lesnicki, Z. Zhang, M. Bonn, M. Sulpizi, E. H. G. Backus, *submitted* **2020**.

[58] S. Kataoka, M. C. Gurau, F. Albertorio, M. A. Holden, S. M. Lim, R. D. Yang, P. S. Cremer, *Langmuir* **2004**, *20*, 1662-1666.

[59] K. Uosaki, T. Yano, S. Nihonyanagi, *J. Phys. Chem. B* **2004**, *108*, 19086-19088.

[60] S. Hosseinpour, F. Tang, F. Wang, R. A. Livingstone, S. J. Schlegel, T. Ohto, M. Bonn, Y. Nagata, E. H. G. Backus, *J. Phys. Chem. Lett.* **2017**, *8*, 2195-2199.

[61] S. J. Schlegel, S. Hosseinpour, M. Gebhard, A. Devi, M. Bonn, E. H. G. Backus, *Physical Chemistry Chemical Physics* **2019**, *21*, 8956-8964.

[62] P. B. Miranda, L. Xu, Y. R. Shen, M. Salmeron, *Phys. Rev. Lett.* **1998**, *81*, 5876.

[63] Z. Yang, A. K. Bertram, K. C. Chou, *J. Phys. Chem. Lett.* **2011**, *2*, 1232-1236.

[64] E. Anim-Danso, Y. Zhang, A. Dhinojwala, *J. Phys. Chem. C* **2016**, *120*, 3741-3748.

[65] A. Abdelmonem, *Atmos. Chem. Phys.* **2017**, *17*, 10733-10741.

[66] A. Tuladhar, Z. A. Chase, M. D. Baer, B. A. Legg, J. Tao, S. Zhang, A. D. Winkelman, Z. Wang, C. J. Mundy, J. J. De Yoreo, H. F. Wang, *J. Am. Chem. Soc.* **2019**, *141*, 2135-2142.

[67] S. Jin, Y. Liu, M. Deiseroth, J. Liu, E. H. G. Backus, H. Li, H. Xue, L. Zhao, X. C. Zeng, M. Bonn, J. Wang, *submitted* **2020**.
